# A HECT Ubiquitin-Protein Ligase as a Novel Candidate Gene for Altered Quinine and Quinidine Responses in *Plasmodium falciparum*

**DOI:** 10.1371/journal.pgen.1004382

**Published:** 2014-05-15

**Authors:** Cecilia P. Sanchez, Chia-Hao Liu, Sybille Mayer, Astutiati Nurhasanah, Marek Cyrklaff, Jianbing Mu, Michael T. Ferdig, Wilfred D. Stein, Michael Lanzer

**Affiliations:** 1 Department of Infectious Diseases, Parasitology, Universitätsklinikum Heidelberg, Heidelberg, Germany; 2 Laboratory for the Development of Agroindustrial and Biomedical Technology (LAPTIAB), Tangerang Selatan, Indonesia; 3 Laboratory of Malaria and Vector Research, National Institute of Allergy and Infectious Diseases (NIAID), National Institutes of Health (NIH), Bethesda, Maryland, United States of America; 4 The Eck Institute for Global Health, Department of Biological Sciences, University of Notre Dame, Notre Dame, Indiana, United States of America; 5 Biological Chemistry, Silberman Institute of Life Sciences, Hebrew University of Jerusalem, Givat Ram, Jerusalem, Israel; Georgia Institute of Technology, United States of America

## Abstract

The emerging resistance to quinine jeopardizes the efficacy of a drug that has been used in the treatment of malaria for several centuries. To identify factors contributing to differential quinine responses in the human malaria parasite *Plasmodium falciparum*, we have conducted comparative quantitative trait locus analyses on the susceptibility to quinine and also its stereoisomer quinidine, and on the initial and steady-state intracellular drug accumulation levels in the F1 progeny of a genetic cross. These data, together with genetic screens of field isolates and laboratory strains associated differential quinine and quinidine responses with mutated *pfcrt*, a segment on chromosome 13, and a novel candidate gene, termed MAL7P1.19 (encoding a HECT ubiquitin ligase). Despite a strong likelihood of association, episomal transfections demonstrated a role for the HECT ubiquitin-protein ligase in quinine and quinidine sensitivity in only a subset of genetic backgrounds, and here the changes in IC_50_ values were moderate (approximately 2-fold). These data show that quinine responsiveness is a complex genetic trait with multiple alleles playing a role and that more experiments are needed to unravel the role of the contributing factors.

## Author Summary

Quinine, a natural product from cinchona bark, has been used in the treatment of malaria for centuries. Unfortunately, a progressive loss in responsiveness of the human malaria parasite *Plasmodium falciparum* to quinine has been observed, particularly in Southeast Asia, where cases of quinine treatment failure regularly occur. To better understand how *P. falciparum* defends itself against the cytotoxic activity of quinine, we have conducted comparative linkage analyses in the F1 progeny of a genetic cross where we assessed the susceptibility and the amount of intracellular accumulation of quinine and of its stereoisomer quinidine. These data identified a novel candidate gene encoding a HECT ubiquitin-protein ligase that might contribute to altered quinine responsiveness. The identification of this novel gene might improve the surveillance of quinine-resistant malaria parasites in the field and aid the preservation of this valuable antimalarial drug.

## Introduction

Quinine, an active ingredient of cinchona bark, is an important drug in the pharmacopoeia against malaria, an infectious disease that causes an estimated 219 million clinical cases and 0.66 million deaths annually [1]. Quinine is used, together with partner drugs, as a second line treatment of uncomplicated malaria and as a first line treatment of malaria in the first trimester of pregnancy [2]. Severe cases of malaria are also frequently treated with quinine, although currently there are better treatment options [2]. Unfortunately, a progressive loss in responsiveness of the human malaria parasite *Plasmodium falciparum* to quinine has been observed, particularly in Southeast Asia [3]–[5] where cases of quinine treatment failure regularly occur, but also in Latin American and Africa [6]–[9]. In spite of quinine's pharmaceutical importance, very little is known about its antimalarial mode of action or the mechanism of resistance. The lack of information, particularly the paucity of genetic markers predictive of quinine resistance, complicates the molecular surveillance of quinine resistant *P. falciparum* strains and jeopardizes efforts to preserve the efficacy of this very valuable drug.

The search for genetic markers of quinine resistance has been complicated by the pleiotropic nature of quinine's mode of action and the complexity of the resistance phenotype. Quinine seems to target endogenous heme detoxification pathways in the parasite's digestive vacuole [10], [11] and it may further block the activity of PfMDR1 [12], [13], a multi-drug resistance transporter predominantly residing at the parasite's digestive vacuolar membrane [14], although the full scope of quinine's molecular targets has yet to be defined. Reflecting the pleitropic mode of action, resistance to quinine seems to be multifactorial. Genetic markers that have been implicated in altered *in vitro* quinine responsiveness include *pfcrt* (chloroquine resistance transporter gene), *pfmdr1* (multi-drug resistance gene), *pfnhe* (sodium/hydrogen ion exchanger gene) and PFD0610w (putative phosphopantothenoylcysteine synthetase gene) [12], [15]–[24]. However, the data linking these genes to altered quinine responsiveness are conflicting and there is evidence suggesting that the genetic background plays an important, hitherto unexplained, role for the ability of any of these genes to confer quinine response variations. For example, a genetic analysis and some, but not all, epidemiological studies found a correlation between the K^76^T polymorphism in *pfcrt* (a mutation indicative of chloroquine resistance in *P. falciparum*) with reduced quinine responsiveness [15], [16], [25], [26], whereas an allelic exchange experiment observed the reverse - an increase in quinine susceptibility when the wild type *pfcrt* allele was replaced by the mutated allele in the *P. falciparum* clone GC03 [27]–[29]. Similarly, the genetic background seems to determine whether mutations within *pfmdr1* and *pfnhe* bring about changes in the susceptibility to quinine [18], [30], [31].

To identify novel factors contributing to reduced quinine responsiveness, we have conducted comparative quantitative trait loci analyses on the susceptibility to quinine and its stereoisomer quinidine and on the initial and steady-state intracellular drug accumulation levels in the F1 progeny of the genetic cross between the *P. falciparum* strains HB3 and Dd2. This approach follows up on the idea of a possible correlation between quinine resistance and reduced intracellular quinine accumulation, as suggested by the fact that *pfcrt*, *pfmdr1* and *pfnhe*, all encode transporters that are thought to contribute to quinine resistance by reducing digestive vacuolar drug concentrations below toxic levels [12], [13], [19], [21], [22], [32]–[34]. Here we describe a novel putative quinine response gene, termed MAL7P1.19 (PF3D7_0704600). MAL7P1.19 encodes a HECT ubiquitin-protein ligase that shares homologies with UFD4 [35], a factor implicated in the ubiquitin fusion degradation pathway and in the Arg/N rule pathway, as shown in *Saccharomyces cerevisiae* [36]

## Results

### Novel QTL on chromosome 7 linked to differential quinine and quinidine responses

**Figure 1:**
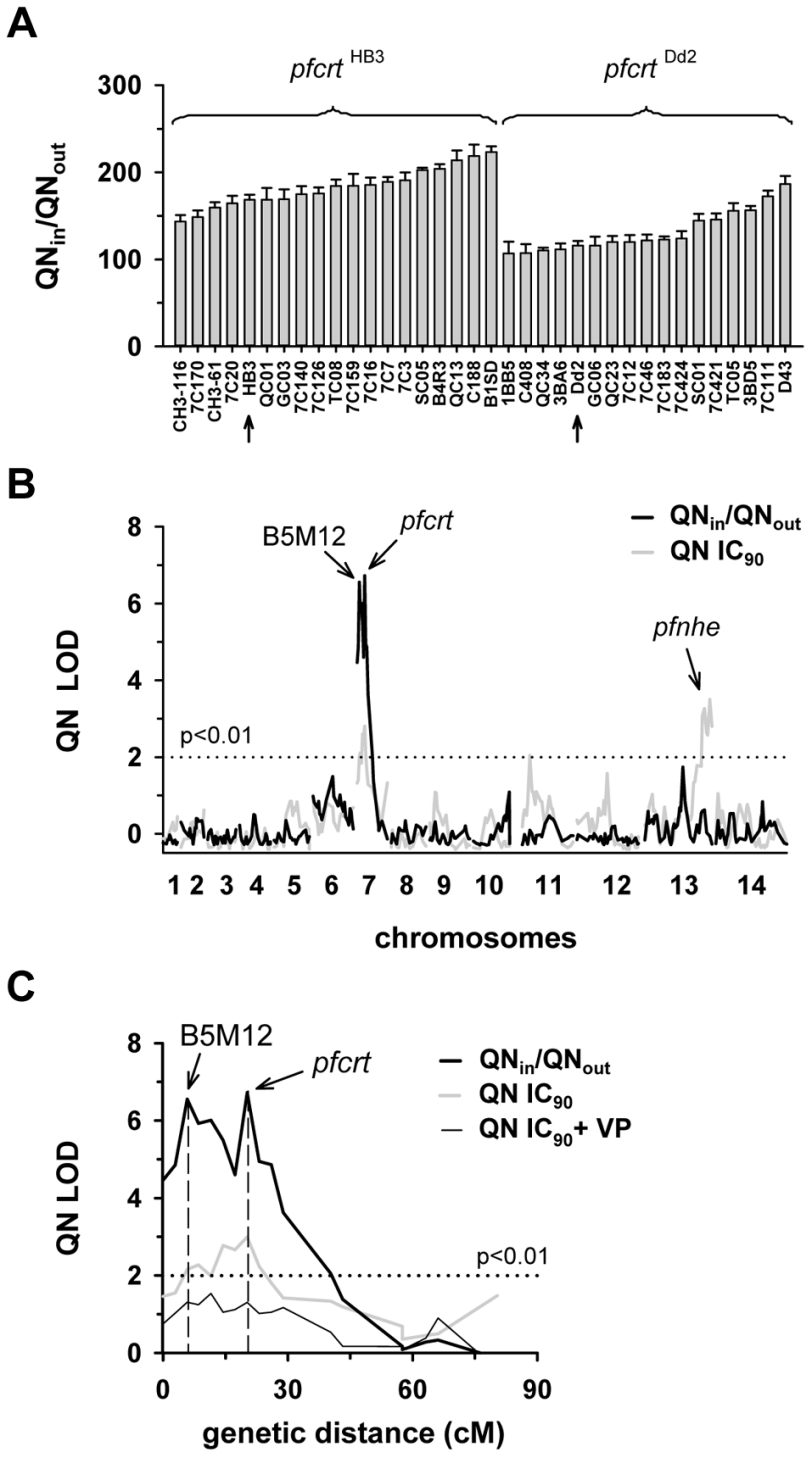
Linkage analyses on quinine responses in the HB3 x Dd2 cross. A. The net intracellular quinine accumulation ratios (given as the ratio of the intracellular versus extracellular quinine concentration, QN_in_/QN_out_) were determined in the F1 progeny from the genetic cross between HB3 and Dd2 and in the two parental strains after 5 min of incubation (initial uptake phase). The names of the progeny and the parental stains are indicated. Progeny containing the wild-type *pfcrt* of HB3 and the polymorphic *pfcrt* of Dd2 are indicated. The means ± SEM of at least 8 biological replicates are shown. B. QTL analyses on the net intracellular quinine accumulation ratios (black line) and the quinine IC_90_ values (grey line) are shown. The logarithm of odds (LOD) scores from the primary scans are shown as a function of genome location. The *pfcrt* and B5M12 loci on chromosome 7 and the bifurcated peak on chromosome 13 are indicated. The dotted line represents the confidence line with P<0.01. C. Enlarged display of the bifurcated peak on chromosome 7. The LOD scores corresponding to i) the quinine accumulation data (thick black line), ii) the quinine IC_90_ values (grey line), and iii) the quinine IC_90_ values determined in the presence of 0.89 µM verapamil (thin black line) are shown. The quinine IC_90_ values in the presence and absence of verapamil were taken from Ferdig et al. 2004 [15]. The analysis of the 25 min quinine accumulation data and the quinine IC_50_ values are shown in the supplementary Figure S2.

**Figure 2:**
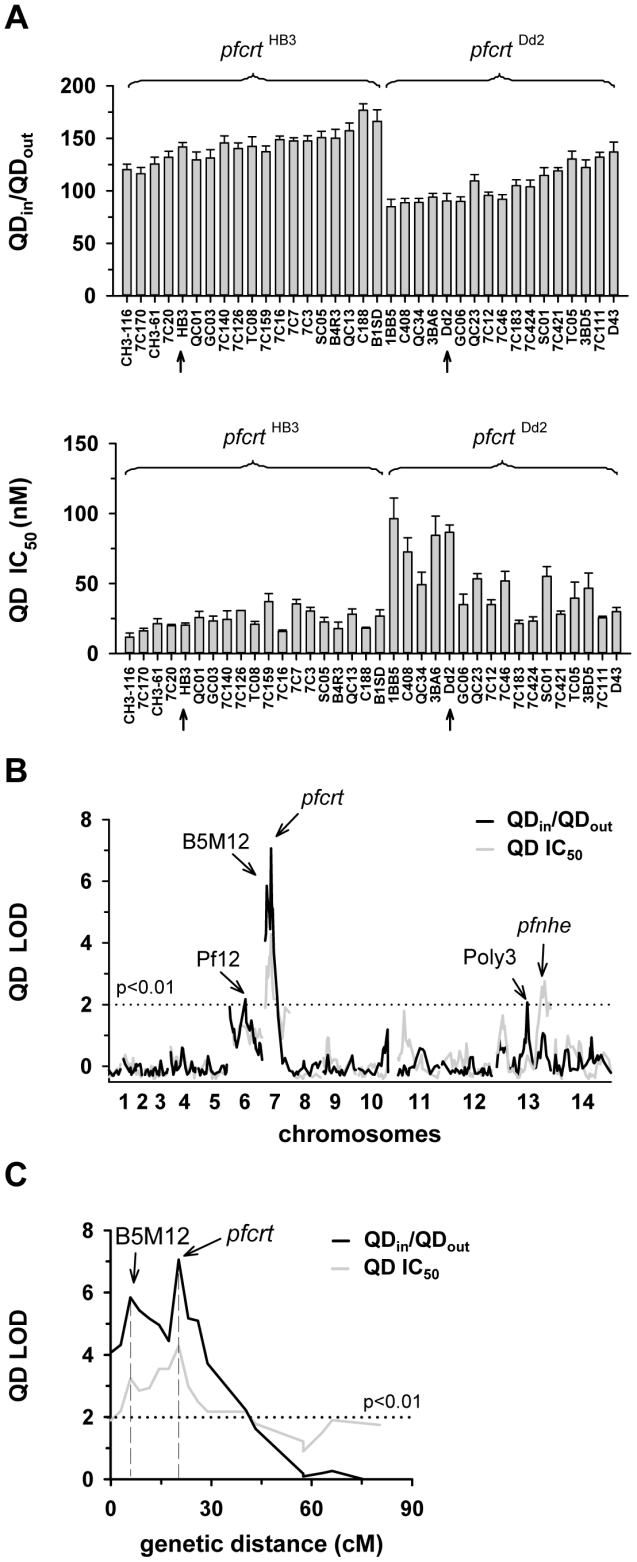
Linkage analyses on quinidine responses in the HB3 x Dd2 cross. A. The quinidine IC_50_ values (upper panel) and the net intracellular quinidine accumulation ratios (QD_in_/QD_out_) (lower panel) were determined in the F1 progeny from the genetic cross between HB3 and Dd2 and in the two parental strains after 5 min of incubation (initial uptake phase). The means ± SEM of at least 8 biological replicates are shown. B. QTL analyses on the net intracellular quinine accumulation ratios (black line) and the quinine IC_50_ values (grey line) are shown. Relevant genetic markers are indicated. C. Enlarged display of the bifurcated peak on chromosome 7, with thick black line and grey line showing the LOD scores for the quinidine accumulation data and the quinidine IC_50_ values, respectively. The analysis of the 25 min quinidine accumulation data are shown in the supplementary Figure S3.

In a previous study we have shown that the *P. falciparum* clone HB3 accumulated with time significantly more [^3^H]-quinine and [^3^H]-quinidine from external concentrations of 40 nM than did the *P. falciparum* clone Dd2 [21]. Time courses of intracellular drug accumulation performed concurrently confirmed this result (supplementary Figure S1). The level of quinine and quinidine accumulation reciprocally correlated with the *in vitro* susceptibility of the two strains to these two drugs, with Dd2 having half maximal inhibitory concentrations (IC_50_ values) for quinine and quinidine three- and four-fold higher than those of HB3 [15] (supplementary Table S1). To identify factors contributing to quinine and quinidine response variations, we performed quantitative trait loci (QTL) analyses on the amounts of quinine and quinidine accumulation at the 5 min (initial uptake phase; Figures 1A and 2A) and 25 min (steady state phase; supplementary Figures S2A and S3A) time points in the published 34 F1 progeny of the HB3 x Dd2 cross and the two parental clones [37] (supplementary Table S1). In addition, we determined the quinidine growth inhibitory concentrations (IC_50_ values) for the F1 progeny and the parental clones (Figure 2A and supplementary Table S1) and analyzed these data by QTL mapping. We further reanalyzed the previously published quinine IC_90_ values and the corresponding IC_50_ values [15] (Figure 1B and supplementary Figure S2A).

The QTL analyses, depicted in the form of the computed LOD scores against the previously described genetic linkage maps of all 14 *P. falciparum* chromosomes [38], identified for both drugs, both assays (accumulation and proliferation assay), both time points, and both IC_50_ and IC_90_ values, a bifurcated peak on chromosome 7 where one finger corresponded to *pfcrt* (20.2 cM) and another, well-separated finger, centered around the marker B5M12 (5.8 cM) (Figures 1B, 1C, 2B, and 2C, supplementary Figures S2B, S2C, S3B, and S3C). The bifurcated peak on chromosome 7 accounted for 59% (64%) and 31% (43%) of the total variance in the quinine (quinidine) accumulation ratios and in the quinine (quinidine) susceptibilities, respectively. As exemplified by the QTL analysis on quinine susceptibility, the contribution of both chromosome 7 peaks was sensitive to verapamil (Figure 1C, thin line), a chemosensitzer and an established inhibitor of PfCRT [20], [22], [39]. The *pfcrt* and the B5M12 peaks are supported by 8 and 17 independent markers, respectively. The bottom of the valley between both peaks is defined by 9 markers and five independent recombination events, in the progeny, between the B5M12 and the *pfcrt* locus (supplementary Table S2). Markers generated as part of this study are listed in supplementary Table S3. Supplementary Figure S4 shows an overview of the B5M12 locus.

The statistical procedure we used for the QTL analysis (see Material and Methods) recorded also the sign of the correlation coefficients between the response variation and the polymorphisms at each genetic locus. For both chromosome 7 peaks, the correlation coefficients were negative for drug accumulation and positive for drug susceptibility (Supplementary Table S4) indicating that it is the presence of the Dd2-inherited loci that is associated with a reduction in quinine and quinidine accumulation and an increase in resistance. Moreover, there is a statistically significant interaction between the *pfcrt* locus and the B5M12 locus (two way ANOVA; P = 0.035 for quinine; P = 0.006 for quinidine), suggesting that the Dd2-inherited B5M12 and *pfcrt* loci co-act in bringing about significant quinine and quinidine response variations.

The QTL analyses further identified a bifurcated peak on chromosome 13 (defined by the markers VAPA and C13M73) that is associated with altered quinine and quinidine susceptibility, but not with altered drug accumulation (Figures 1B and 2B, supplementary Figures S2B, and S3B, and Supplementary Table S4). The bifurcated peak on chromosome 13 explains 35% and 27% of the total variance in quinine and quinidine susceptibility observed in the F1 progeny. It needs to be of Dd2 origin to confer an increase in resistance. In addition to the QTLs on chromosomes 7 and 13, no further QTLs rose above the confidence line in the genetic scans using the quinine and quinidine IC_50_ or IC_90_ values or the quinine accumulation data (p<0.01, Figures 1B and 2B and supplementary Figure S2B). For the quinidine accumulation data, two additional QTLs were observed: PF12 on chromosome 6 (51.7 cM) and Poly3 on chromosome 13 (107.3 cM) (Figure 2B, supplementary Figure S3B, and Supplementary Table S4).

In secondary scans, we separately analyzed the progeny that carried the wild type *pfcrt* allele and the progeny that carried the mutated *pfcrt* allele, thereby eliminating the contribution of *pfcrt* to drug response variations [40]. The secondary scans again identified the B5M12 locus with both drugs and for both time points at which drug accumulation levels were determined (Supplementary Table S4). In addition, the secondary scans revealed additional minor QTLs; some were shared between quinine and quinidine, including *pfmdr1* (69.2 cM), B5M86 (60.2 cM) and C5M2 (2.6 cM) on chromosome 5 and MEF1 (32.6 cM) and Poly3 (107.3 cM) on chromosome 13. The loci *pfmdr1*, B5M86 and C5M2 contributed to increased quinine and quinidine accumulation, whereas the other three loci were associated with reduced drug accumulation, when inherited from Dd2 (Supplementary Table S4). Other QTLs were specific for quinidine, including C9M43 on chromosome 9 (0 cM) and TPI and C14M75 on chromosome 14 (123.4 cM and 9.6 cM).

Secondary scans performed on the IC_50_ values revealed additional QTLs that contributed to both altered quinine and quinidine susceptibility, including BM75 and BM103 (31.7 cM and 100.5 cM) on chromosome 6. Other minor QTLs seem to confer stereoisomeric responses, such as B5M4 (23.1 cM) on chromosome 6 and C13M73 (178.8 cM) on chromosome 13, which are specific for quinine, and B7M14 (51.6 cM) on chromosome 10 and AG15 (60.3 cM) on chromosome 11, which are specific for quinidine (Supplementary Table S4). Minor QTLs and QTLs identified in secondary scans were not investigated further.

### *pfnhe* expression levels do not affect quinine and quinidine accumulation levels

**Figure 3:**
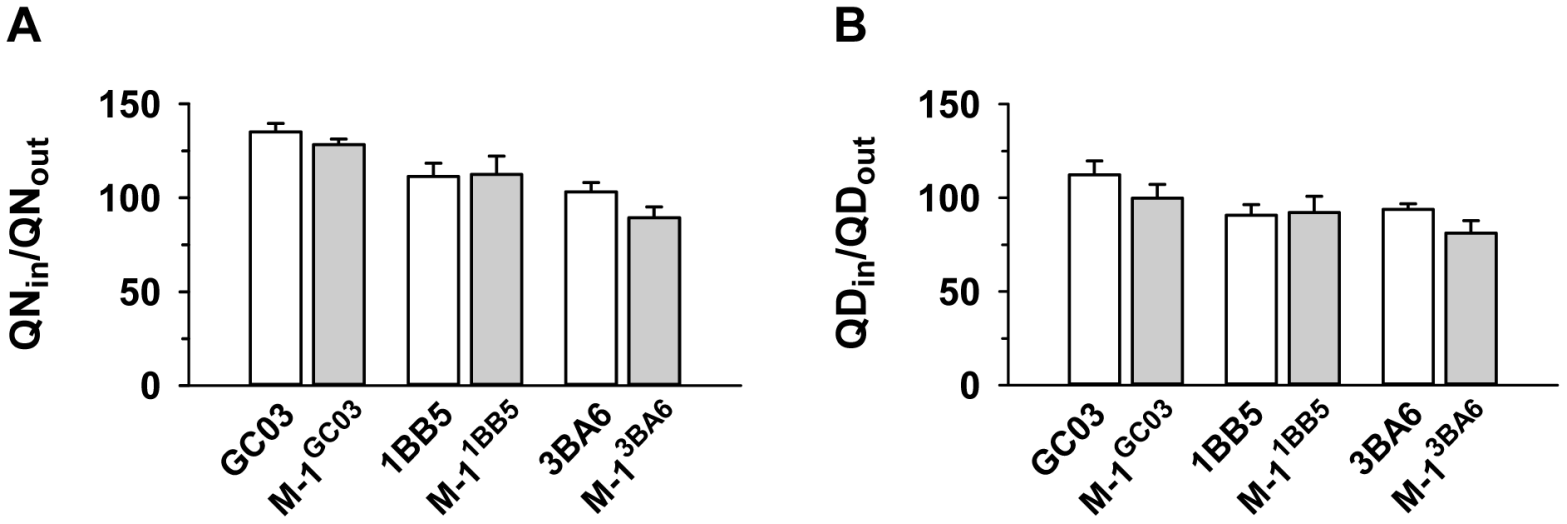
Effect of *pfnhe* on quinine and quinidine accumulation. Quinine (A) and quinidine (B) accumulation ratios (at 5 min) in different *pfnhe* expression mutants [18] and in the corresponding parental strains. The means ± SEM of at least 8 biological replicates are shown.

The segment of chromosome 13 that is associated with differential quinine and quinidine susceptibility, but not with altered drug accumulation levels, contains *pfnhe*, a gene that some, but not all, studies have implicated in altered quinine responsiveness and which is thought to affect intracellular quinine partitioning [15], [16], [25], [29]. In a recent study, it has been shown that genetically-engineered *pfnhe* mutants with down-regulated *pfnhe* expression levels displayed decreased quinine IC_50_-values, dependent upon the genetic background of the strain [18]. The two *pfnhe* mutants that revealed increased quinine susceptibility were M-1^1BB5^ and M-1^3BA6^, whereas M-1^GC06^ did not show this phenotype [18]. For each of the three *pfnhe* mutants and their corresponding unmutated progenitor lines, we measured the accumulation of quinine and quinidine at the 5 min time point (Figures 3A and B). In none of the cases was there a statistically significant difference in drug accumulation as between the wild type and down-regulated *pfnhe* mutant under the conditions employed in this study.

### Effect of the B5M12 locus on quinolone response variations

**Figure 4:**
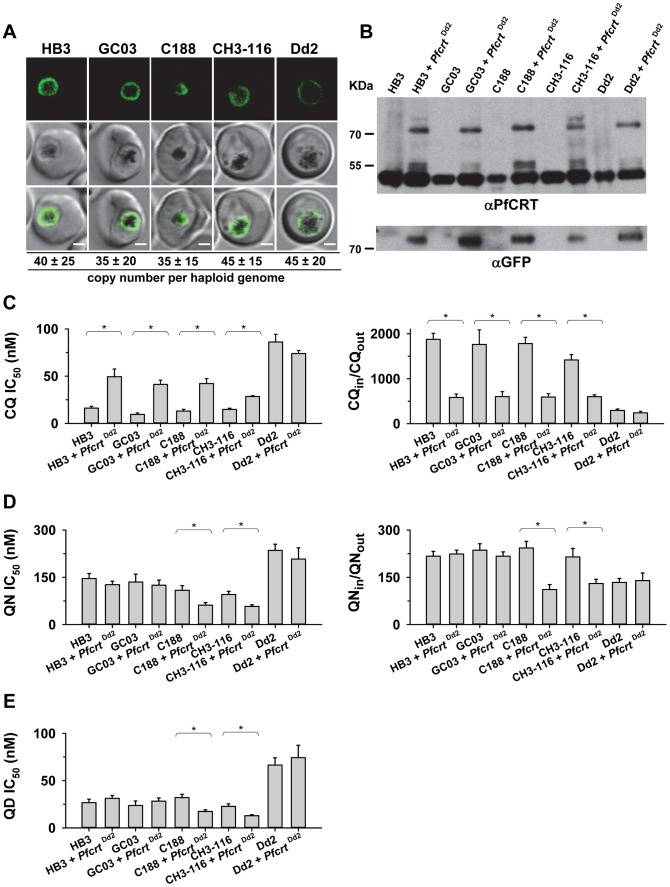
Functional association between the B5M12 locus and mutant PfCRT in conferring quinine and quinidine response variations. A. Live cell images of the *P. falciparum* strains indicated expressing an episomal copy of the Dd2 form of *pfcrt* fused to the GFP coding sequence. Fluorescence is located at the digestive vacuolar membrane, consistent with previous reports [69]. Bar, 2 µM. The copy number of the plasmid per haploid genome is indicated. The means ± SEM of at least 6 biological replicates are shown. The copy numbers are not statistically different in the transfectants. B. Western analysis using an antiserum specific to PfCRT. Total protein from 6×10^6^ parasites each were size-fractionated by SDS PAGE on a 4–12% gradient gel, transferred to a polyvinylidene difluoride membrane, and analyzed using an antiserum specific to PfCRT (αPfCRT, dilution 1∶1000; upper panel) and GFP (αGFP, dilution 1∶1000; lower panel). The molecular weight of the endogenous PfCRT is 48.7 kDa and that of the episomally expressed PfCRT/GFP fusion protein is 75.6 kDa. C. Chloroquine IC_50_ values (left panel) and accumulation ratios at the 20 min time point (right panel) in transfected parasite lines and the corresponding parental strains. D. Quinine IC_50_ values (left panel) and accumulation ratios at the 20 min time point (right panel). E. Quinidine IC_50_ values. The means ± SEM of at least 10 independent determinations are shown in parts D to E. *, P<0.01. The genetic backgrounds of the parasite lines with regard to the relevant chromosome 7 and 13 markers is compiled in Table 1.

To further examine the contribution of the B5M12 locus to differential quinine and quinidine responses, we selected three progeny (GC03, CH3-116 and C188) that harbor the wild type *pfcrt* allele but which differ with regard to the B5M12 locus. GC03 contains the wild type HB3 B5M12 variant, whereas CH3-116 and C188 inherited the B5M12 locus from Dd2 (Table 1). The three progeny and the two parental clones HB3 and Dd2 were transfected with a vector expressing the Dd2 *pfcrt* variant fused in frame with the coding sequence of the green fluorescence protein (GFP). The vectors were maintained at approximately 40 copies per haploid genome, with no significant differences between the transfectants (Figure 4A). In all cases, the PfCRT/GFP fusion protein was expressed and localized at the membrane of the parasite's digestive vacuole, as determined by live cell fluorescence microscopy (Figure 4A). Western analyses using an antiserum specific to PfCRT confirmed the expression of the 75.6 kDa PfCRT/GFP fusion protein in the transfectants (Figure 4B). However, the amount of protein was lower than that of the endogenous PfCRT (48.7 kDa, Figure 4B).

**Table 1:** Relevant genotypes of selected progeny and the parental strains Dd2 and HB3.

		Strains							
Marker	Chr.	Dd2	HB3	C188	CH3-116	D43	GC03	TC05	7C111
B5M12	7	D	H	D	D	H	H	H	H
pfcrt	7	D	H	H	H	D	H	D	D
VAPA	13	D	H	H	H	H	D	D	D
C13M73	13	D	H	H	H	H	D	D	D

D and H indicate inheritance of the marker from Dd2 and HB3, respectively. Chr., chromosomal location of the marker indicated.

We then determined the responses of the transfectants to quinine, quinidine, and chloroquine - the latter drug serving as a control. In all transfected lines, with the exception of the transfected Dd2 line, there was a significant increase in chloroquine IC_50_ values and, associated therewith, a substantial reduction in chloroquine accumulation ratios, as compared to the corresponding parental strains (P<0.01; Figure 4C). This finding indicates that the episomally expressed Dd2 *pfcrt* variant is functional and confers a dominant positive phenotype with regard to chloroquine resistance, although the degree of resistance fell short of that of Dd2, possibly due to the low expression level of the episomally encoded *pfcrt* gene [41] and/or because of a weakened chloroquine transport activity of the GFP-extended PfCRT protein.

In comparison to chloroquine where all the *pfcrt* transfectants, except for that of Dd2, differentially responded to the drug, the response variations observed for quinine and quinidine were multifarious. A reduction in quinine accumulation levels was only found in CH3-116 and C188, the two progeny harboring the B5M12 locus from Dd2, and not in GC03 or HB3 that both possess the wild type HB3 B5M12 locus (Figure 4C; Table 1). This finding is consistent with the QTL analysis that identified the B5M12 and the *pfcrt* locus as the two principal and co-acting contributors to differential quinine accumulation ratios in the HB3 x Dd2 cross. Interestingly, the reduction in quinine accumulation ratios did not correlate with an increase in quinine IC_50_ values as one would have expected by analogy with chloroquine. Instead, CH3-116 and C188 became significantly more quinine and also more quinidine-sensitive when episomally expressing the Dd2 *pfcrt* variant, with the IC_50_ values dropping to 54–60% of those of the corresponding parental strains (Figures 4D and C). No changes in susceptibility to the two enantiomers were observed in the HB3, GC03, and Dd2 background. In this context it should be noted that the B5M12 and the *pfcrt* locus jointly contribute only a third to the total variance in quinine and quinidine susceptibility in the HB3 x Dd2 cross. Another third is attributed to the bifurcated peak on chromosome 13 (see above) and a final third to various minor QTLs. Both CH3-116 and C188 inherited the respective chromosome 13 domains from HB3 (Table 1) suggesting that, while the presence of both the B5M12 and the *pfcrt* locus from Dd2 is sufficient to reduce intracellular quinine accumulation, this does not suffice to increase the level of resistance without additional other QTLs being also of the Dd2 type, such as the two chromosome 13 loci. That expression of the mutant *pfcrt* gene in certain genetic backgrounds results in increased, and not in reduced, susceptibility to quinine and quinidine has also recently been observed [27], [42]. It is explained by the PfCRT-mediated drug transport enhancing the encounter of the drug with targets outside the digestive vacuole [42]. The contributing genes in the chromosome 13 QTLs might be such targets (see discussion). Transfecting the strains with a vector expressing the wild type *pfcrt* fused to GFP had no effect on chloroquine or quinine responses (supplementary Figure S5).

### Delineation of the B5M12 locus

The B5M12 locus consists of 33 annotated genes of which four are for t-RNAs (Supplementary Figure S4). We undertook a search among the remaining 29 annotated gene sequences to identify polymorphisms that might correlate with the changes in quinine and quinidine responses. To this end, we analyzed available genome sequence databases for HB3 and Dd2 and, in addition, amplified and sequenced the respective open reading frames from both parental clones. For 7 of these annotated genes, we could find no polymorphic differences between HB3 and Dd2. For 4 of them, we failed to obtain sequence information. Those annotated genes for which we found polymorphisms (either as codon replacements or as length polymorphisms) are listed in supplementary Table S5. In total we identified 109 polymorphisms in the B5M12 locus between HB3 and Dd2. We selected 92 polymorphisms in 20 genes for further analysis. Six genes harboring a single conservative amino acid replacement were not followed through.

**Figure 5:**
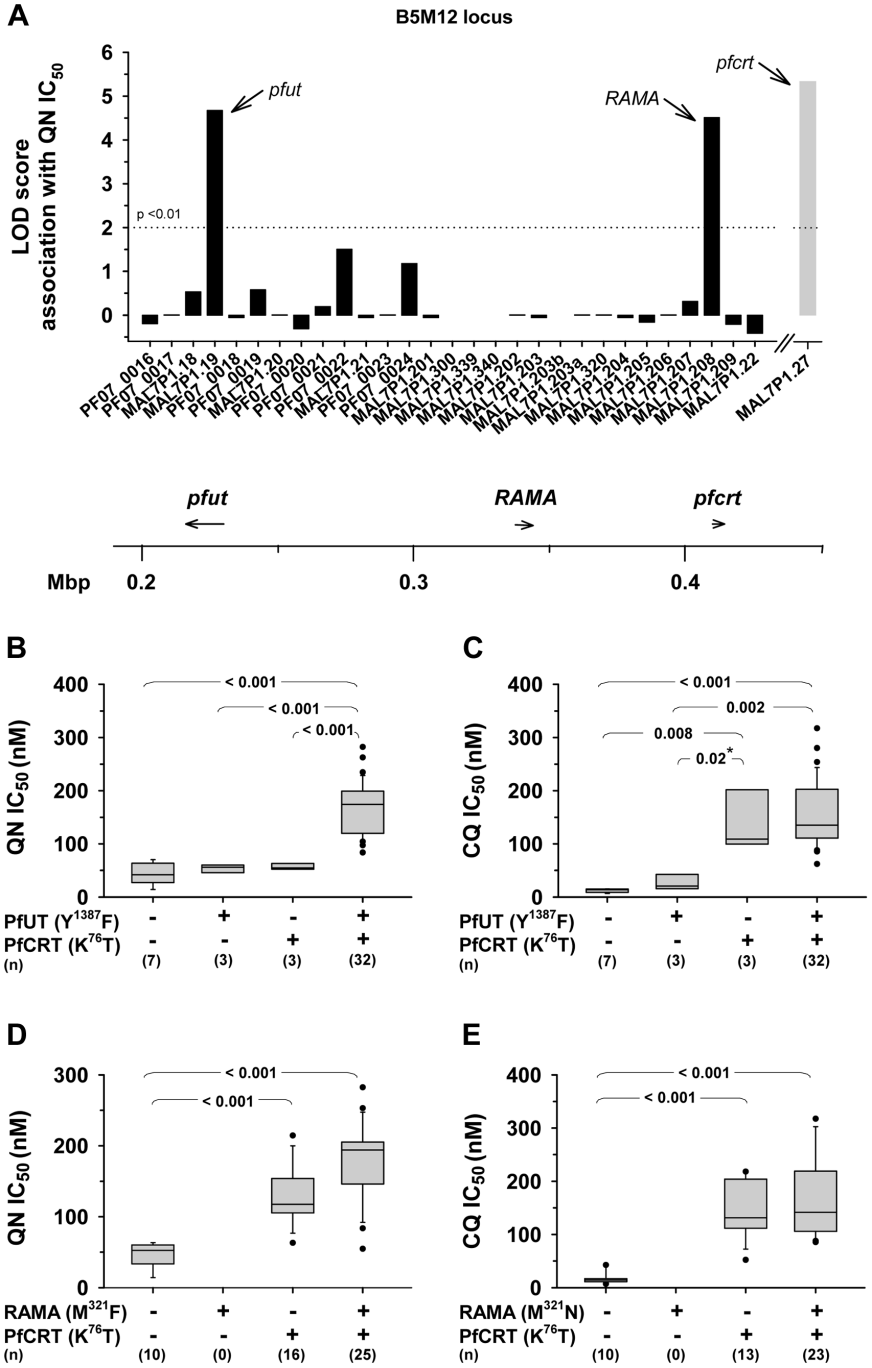
Delineation of the B5M12 locus. A. Linkage analysis on quinine IC_50_ values with polymorphic annotated genes in the B5M12 locus in 50 field isolates and laboratory strains. The LOD score of *pfcrt* (grey bar), which resides outside the B5M12 locus, is shown for comparison. Gaps indicate genes that were not included in the analysis. The confidence line is indicated. Arrows point towards *pfut*, RAMA and *pfcrt*. The location (according to the 3D7 reference sequence) and orientation of pfut, RAMA and pfcrt on chromosome 7 is indicated in the schematic drawing below. B and C. Parasites were grouped according to their haplotypes with regard to *pfcrt* and the HECT ubiquitin-protein ligase gene (*pfut*) and analyzed as a function of the quinine IC_50_ values (B) and the chloroquine IC_50_ values (C). D. and E. Parasites were grouped according to their haplotypes with regard to *pfcrt* and RAMA and analyzed as a function of the quinine IC_50_ values (D) and the chloroquine IC_50_ values (E). Statistical significance between different parasite groups is indicated. *, Fisher's LSD test, in all other cases one way ANOVA test. The quinine and chloroquine IC_50_ values were taken from Mu et al. (2003) [43].

A study by Mu et al (2003) has recorded the IC_50_ values for quinine and chloroquine for a large number of field isolates and laboratory strains of *P. falciparum* from different geographic origin [43]. From this collation we selected 26 strains from Southeast Asia, 12 strains from Africa, 10 strains from Latin America, one strain from Papua New Guinea and one strain of unknown origin. In DNA extracted from these 50 strains, we identified the specific polymorphism in the annotated genes selected within the B5M12 locus, as well as in *pfcrt* and *pfmdr1* (supplementary Table S5). Ten of the strains had a wild type *pfcrt* and 40 of the strains had a mutated *pfcrt* allele (as defined by the K^76^T polymorphism). The polymorphisms in a strain were then correlated with the IC_50_ values for quinine or chloroquine. A score was obtained equivalent to a LOD score over the whole set of polymorphisms. In the LOD score presentations, shown as the histogram of Figure 5A, the bars indicate the peak LOD score at each annotated gene. A horizontal line at height zero indicates no polymorphism at this locus, while a gap indicates a gene that escaped analysis or was not selected for further analysis. Two genes were associated with a major peak in LOD score for the quinine IC_50_ values. The left hand (upstream) peak is at a single gene locus identified as MAL7P1.19 (PF3D7_0704600), a gene encoding a putative HECT ubiquitin-protein ligase (originally annotated as a putative ubiquitin transferase) [35] and, henceforth termed *pfut*. Particularly, a set of five amino acid replacements at positions 1375 (N to S), 1387 (Y to F), 1401 (E to D), 1406 (G to C), and 1407 (E to D) were significantly associated with altered quinine responsiveness. The right hand (downstream) peak corresponds to the RAMA gene (Rhoptry Associated Membrane Antigen; MAP7P1.208). We repeated the analysis, omitting a random five of the 50 strains and obtained a very similar LOD score profile with the same major peaks at the *pfut* gene and at RAMA. We repeated the 5 of 50 random omissions procedure another four times and, in each case, received much the same profile (data not shown).

To assess which of the two genes, *pfut* or RAMA, determines reduced quinine susceptibility, we grouped the strains according to their haplotypes with regard to *pfcrt* and *pfut* or *pfcrt* and RAMA. We considered all *pfut* genes encoding a Y^1387^F substitution and all RAMA encoding a M^321^F substitution as mutant. A correlative box plot analysis of these groups with the IC_50_ values for quinine and chloroquine revealed clear distinctions between the two drugs (Figure 5). For quinine, *pfcrt* and *pfut* must both be present in the mutated form to obtain a significant increase in the IC_50_ value (Figure 5B), whereas for chloroquine it is sufficient that only *pfcrt* is present as the mutant, while further mutation at the *pfut* gene does not increase the IC_50_ value significantly (Figure 5C). The segment on chromosome 13 was not considered in this analysis.

For the RAMA gene, there is no strain that has a mutant copy of this gene together with a wild type *pfcrt* gene, so that a full statistically valid grouping analysis, similar to that performed for the *pfut* gene, could not be done. Nevertheless, it is clear that, in contrast to the case for the *pfut* gene, there is not a significant increase in quinine IC_50_ value (p = 0.10) when, in the background of mutant *pfcrt*, the wild type (HB3) form of RAMA is replaced by the mutant form (compare columns 3 and 4 of Figure 5D). Similarly, RAMA had no statistically valid effect on chloroquine IC_50_ values (Figure 5E). These data would suggest that RAMA does not contribute to reduced quinine responsiveness. A secondary scan among strains containing a mutant *pfcrt* revealed a non-significant association of reduced quinine susceptibility with *pfmdr1* (data not shown).

### Polymorphisms within *pfut* and *pfcrt* are co-selected

**Figure 6:**
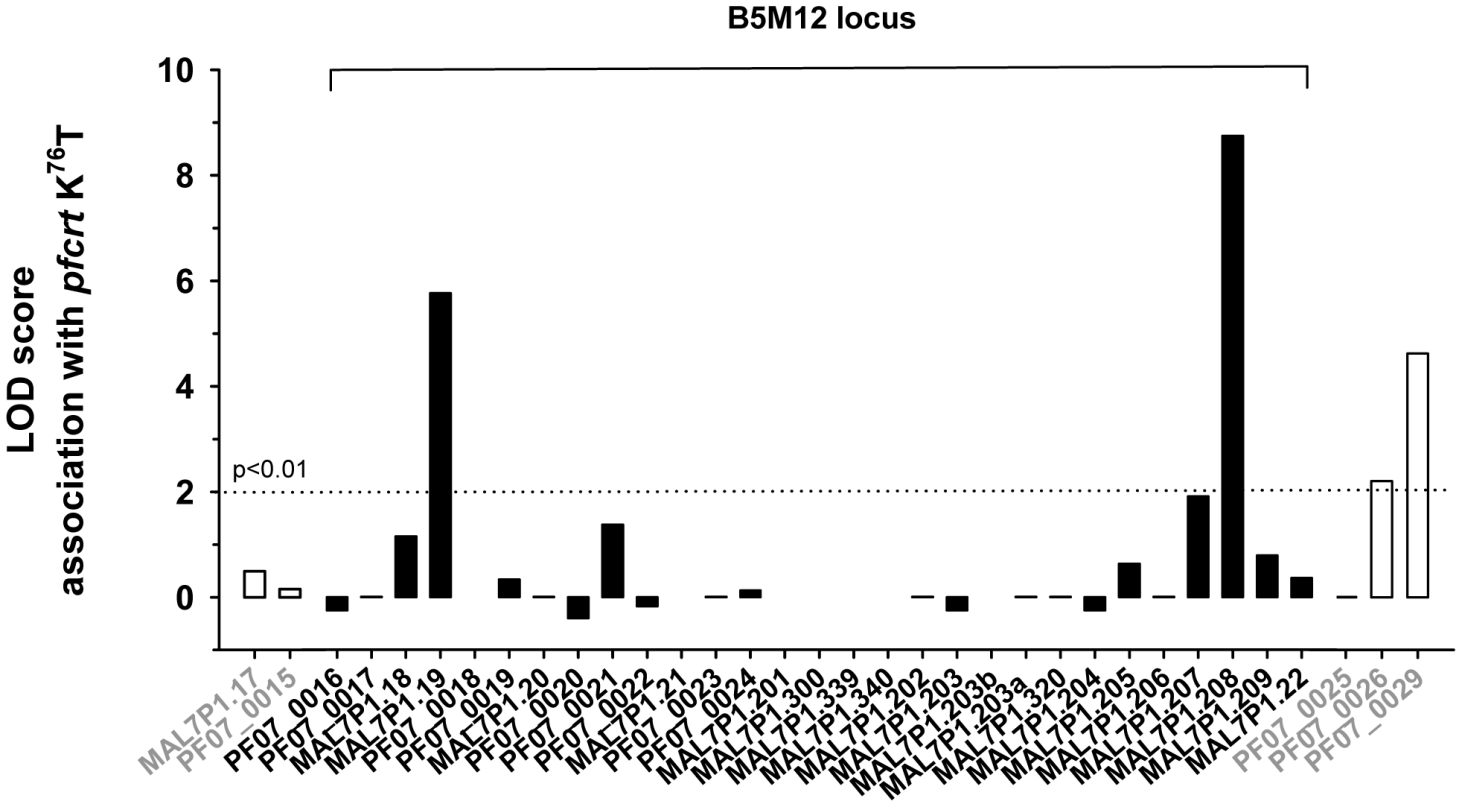
Association of polymorphic genes within the B5M12 locus (black bars) and flanking regions (white bars) with mutant *pfcrt* as defined by the K^76^T replacement. The confidence line is indicated.

Previous studies have identified a region around *pfcrt* on chromosome 7 that is conserved in many chloroquine resistant field isolates and laboratory strains and which co-segregates with *pfcrt* [44]–[46]. To assess whether the genes of the B5M12 locus segregate independently of, or together with *pfcrt*, we correlated, in the 50 *P. falciparum* strains, the presence of mutated *pfcrt* with the polymorphic markers identified in the B5M12 locus and in genes flanking this chromosomal domain. The putative *pfut* gene, RAMA and the genes downstream of the B5M12 locus, towards the *pfcrt* locus (including PF07_0026 and PF07_0029), were significantly associated with mutated *pfcrt*, but this is not the case for the 22 genes in the B5M12 locus that lie between the putative *pfut* gene and RAMA (Figure 6). This finding suggests that the putative *pfut* gene is co-selected with *pfcrt* and does not co-segregate with *pfcrt* due to physical linkage. This cannot be said of RAMA. RAMA seems to be part of the low variability region that is conserved in many *P. falciparum* strains harboring a mutant *pfcrt* and which co-segregates with *pfcrt*.

**Figure 7:**
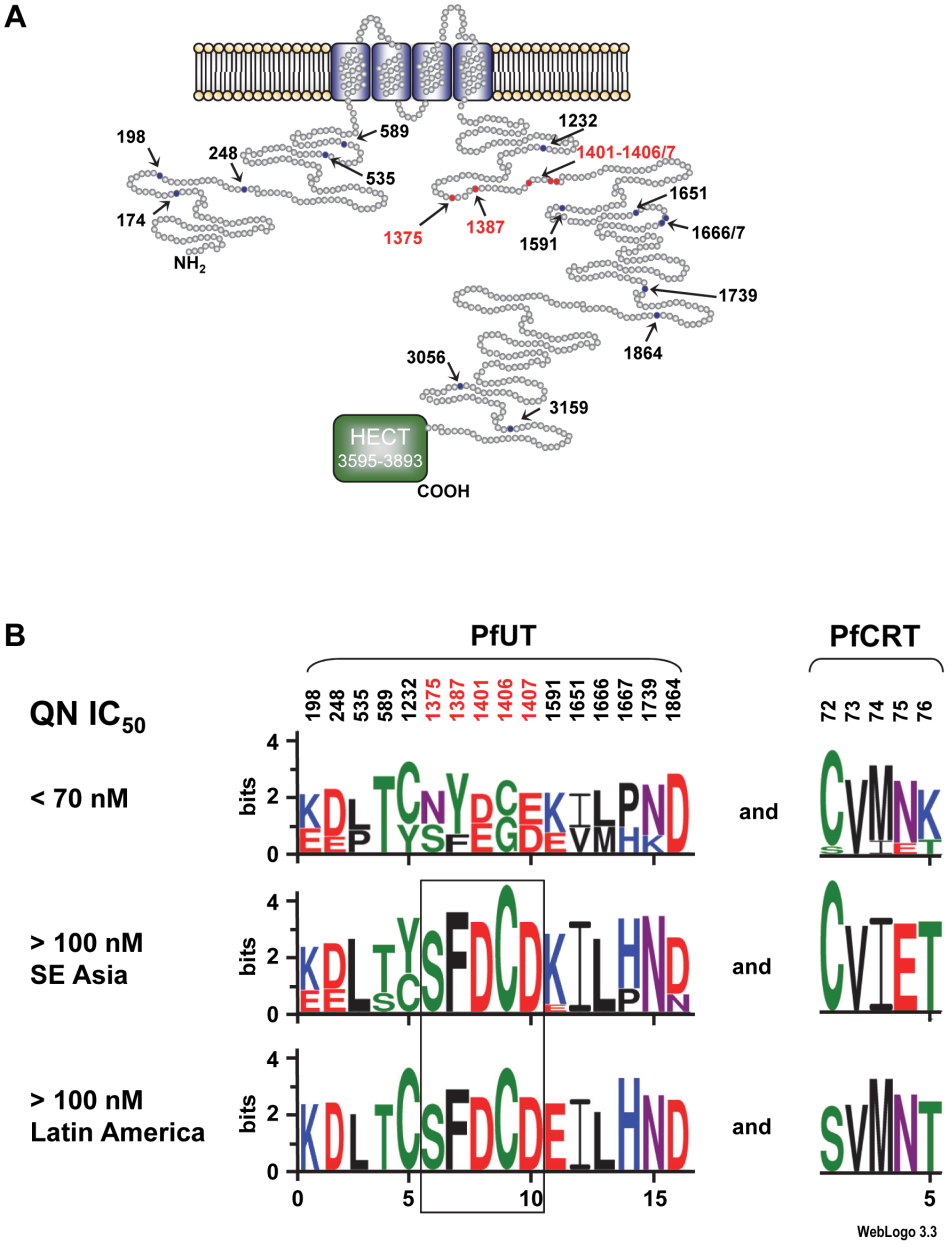
Schematic representation of the HECT ubiquitin-protein ligase (PfUT). A. Topological model of the HECT ubiquitin-protein ligase. The protein consists of 3893 amino acids and has a predicted molecular mass of 460,420 kDa. The protein has four putative transmembrane domains and a catalytic domain (HECT domain) that characterizes the protein as a member of the ubiquitin ligase family. Note that the predicted size of the HECT domain depends on the search engine used. The model shows the HECT domain as predicted by PFAM. Polymorphic residues analyzed in this study are indicated. Red highlights conserved polymorphisms. B. The 50 *P. falciparum* field isolates and laboratory strains depicted in supplementary Table S5 were grouped with regard to their quinine IC_50_ values. Parasites with IC_50_ values exceeding 100 nM were subsequently grouped according to their geographic origin. The sequence logos show the degree of conservation within polymorphic sites within PfUT and PfCRT. The height of each letter is proportional to the frequency of amino acids in each position. The quinine and chloroquine IC_50_ values were taken from Mu et al. (2003) [43]. The number of strains included in each group is as follows: Latin America (n = 9), Southeast Asia (n = 23), and strains with IC_50_ values <70 nM (n = 13). The two African strains with IC_50_ values >100 nM for which sequence data on PFUT were available shared the conserved set of polymorphic amino acids (see supplementary Table S5). Two Africans stains could not be grouped due to incomplete sequence information regarding PfUT and one strain from Latin America that had a quinine IC_50_ value of 84 nM was also not included in the analysis.

Co-selection of *pfut* with *pfcrt* is further supported by the presence of conserved polymorphisms within the HECT ubiquitin ligase in *P. falciparum* strains that carry different mutant *pfcrt* haplotypes. Including the tyrosine to phenylalanine replacement at position 1387, we identified 19 single amino acid polymorphisms and 4 length polymorphisms within PfUT (Figure 7A and supplementary Table S5). Grouping the 50 field isolates and laboratory strains according to their geographic origin and their quinine IC_50_ values, revealed conserved polymorphisms in the *pfut* gene, which are present in strains from Latin America, Africa, and Southeast Asian with quinine IC_50_ values exceeding 100 nM (Figure 7B). Note that the strains from Latin America and from Asia have experienced different drug selection histories and, accordingly, possess distinct *pfcrt* haplotypes [38], [42].

### PfUT localizes to the ER/Golgi complex

The gene *pfut* encodes a protein of 3893 amino acids that is predicted to have four transmembrane domains and to belong to a subfamily of enzymatically active ubiquitin-protein ligases that contain an N-terminal armadillo-like fold implicated in substrate binding and a C-terminal HECT domain (homologous to the E6-AP carboxyl terminus) (Figure 7A) [35]. As shown in other systems, the HECT domain catalyzes ubiquitination. It accepts ubiquitin from a charged E2 conjugating enzyme via a cysteine thioester intermediate and subsequently transfers the ubiquitin to a substrate protein or to the growing end of a multiubiquitin chain [47]. The ability to bind ubiquitin distinguishes HECT domain ubiquitin ligases from other types of E3 ligases that do not form a transient intermediate with ubiquitin and, instead, facilitate the reaction between E2 and the substrate protein by bringing both in close proximity [47].

**Figure 8:**
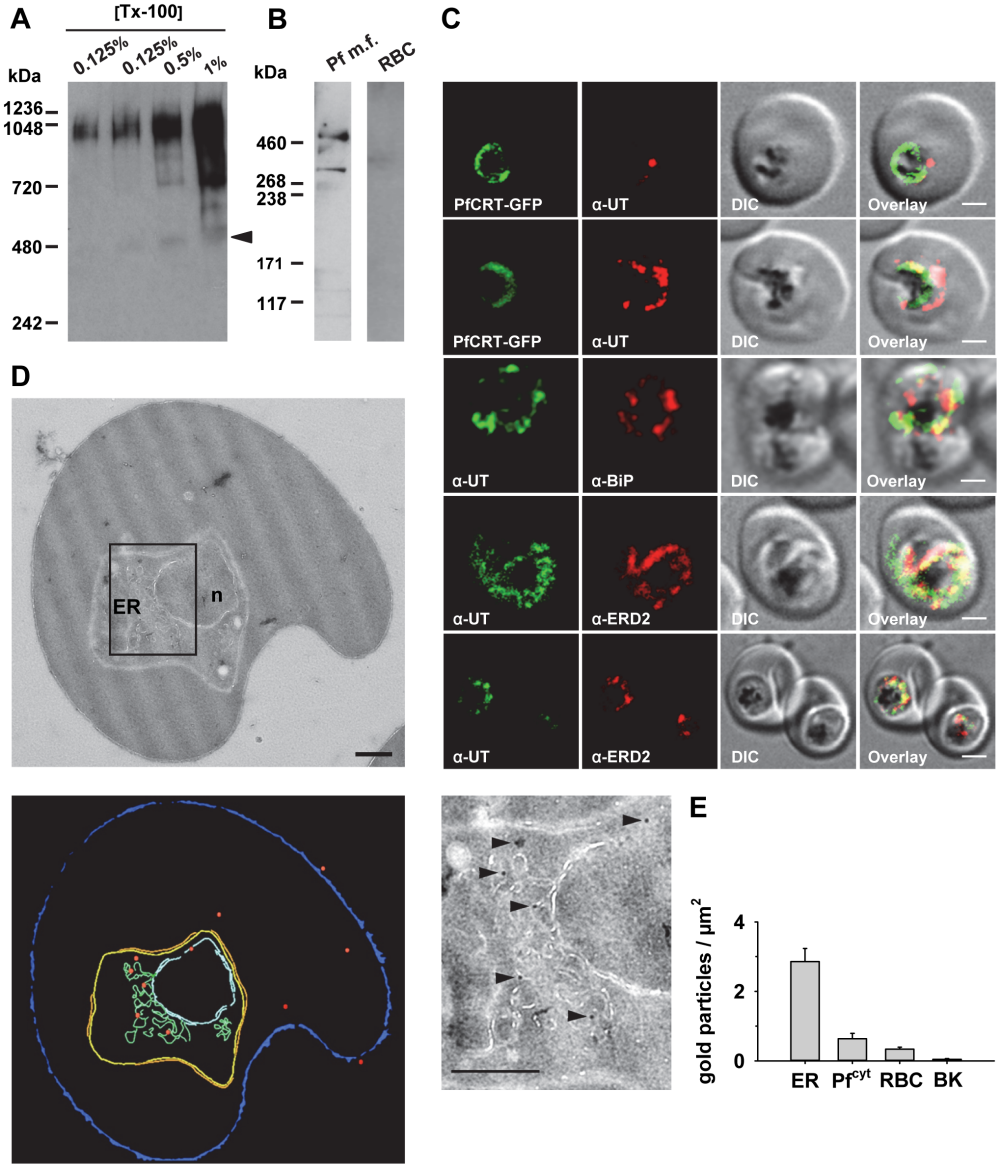
HECT ubiquitin-protein ligase (PfUT) localizes to the ER/Golgi complex. A. PfUT forms high molecular weight complexes under native conditions. Isolated purified trophozoites were extracted with increasing concentrations of Triton X-100. Extracted proteins were size fractionated using a native blue gel. PfUT was subsequently detected using a rabbit peptide antisera (1∶1000). At low concentrations of Triton X-100, high molecular weight species of >1 MDa were visible, whereas at high Triton X-100 concentrations a species corresponding to the predicted molecular weight of PfUT was detected (arrowhead). B. Membrane proteins extracted with Triton X-114 from purified and isolated trophozoites were size fractionated by SDS-PAGE and analyzed using a rabbit antisera specific to the N-terminal domain of PfUT (1∶1000). PfUT is detectable as a species of 460 kDa under denaturing and reducing conditions. Total membrane proteins from uninfected erythrocytes (RBC) were used as a control. C. Subcellular localization of PfUT. *P. falciparum*-infected erythrocytes at the trophozoite stage were fixed and analyzed by immunofluorescence assays using antisera to the ER marker BiP (rabbit, 1∶1000), the Golgi marker ERD2 (rat, 1∶500), and the N- (panels 1 and 5, rabbit, 1∶3000; panel 3, mouse, 1∶2000) and C-terminal domains of PfUT (panels 2 and 4, rabbit, 1∶3000). Panel 1 shows a late ring stage parasite, the other panels show trophozoites. GFP fluorescence was detected, by confocal fluorescence microscopy, in parasites expressing episomally a PfCRT/GFP fusion protein. The different antisera raised against PfUT showed comparable results. Bar, 2 µm. D. Subcellular localization of PfUT by immunoelectron microscopy. The upper panel shows a representative micrograph of a *P. falciparum*-infected erythrocyte preserved by high-pressure freezing and freeze-substitution, and immunolabelled with a rabbit antiserum specific to the N-terminal domain of PfUT (1∶100) coupled to 10 nm protein A colloidal gold. The lower panel shows the surface rendered view of the micrograph, with red dots representing gold grains. Insert: Magnification of boxed section in micrograph. Arrowheads point towards gold label. n, nucleus; fv, food vacuole. Scale bar in D and E, 500 nm. E. Quantification of immuno EM results. The distribution of gold grains was determined in 15 micrographs and analyzed according to their subcellular localization per µm^2^. Gold grains were significantly more present in areas of ER/Golgi complex (ER) than in other subcellular compartments, including the cytoplasm of the parasite (Pf^cyt^), the cytoplasm of the host cell (RBC), and non cellular background (BK) (P<0.001).

Antibodies raised against the N- and the C-terminal domain of PfUT identified a high molecular protein complex of >1 MDa in extracts prepared from isolated parasites solubilized with increasing concentrations of Triton X-100 (Figure 8A). Under reducing and denaturing conditions, a protein of 460 kDa was identified in total membrane fractions extracted with Triton X-114 (Figure 8B), consistent with the predicted molecular weight of this protein. Immunofluorescence microscopy partially co-localized PfUT with the ER marker BiP and the Golgi marker ERD2, but not with PfCRT (Figure 8C). Quantitative immunoelectron microscopy confirmed a predominant localization of PfUT at the ER/Golgi complex (Figure 8D and E).

### Overexpression of the PfUT HECT domain causes quinine response variations

**Figure 9:**
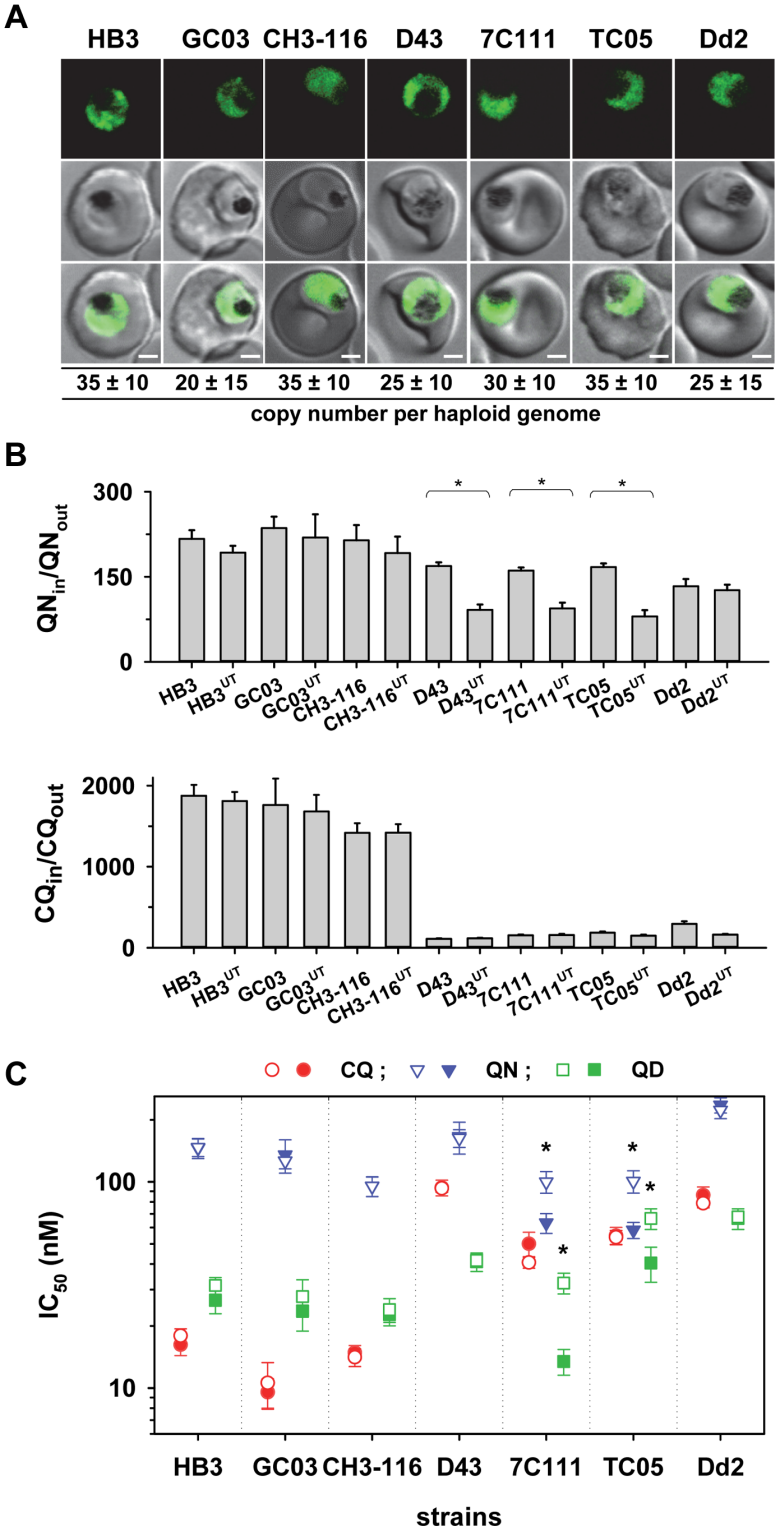
Overexpression of the HECT domain of PfUT confers quinine and quinidine response variations in appropriate genetic backgrounds. A. Live cell images of the *P. falciparum* strains indicated expressing an episomal copy of the catalytic domain of PfUT fused to GFP. The fluorescence signal is located in the parasite's cytoplasm. Bar, 2 µM. The copy number of the plasmid per haploid genome is indicated. The means ± SEM of at least 6 biological replicates are shown. The copy numbers are not statistically different in the transfectants. B. Quinine (upper panel) and chloroquine (lower panel) accumulation ratios at the 20 min time point in the transfected parasite lines and the corresponding parental strains. The means ± SEM of at least 10 biological replicates are shown. *, P<0.001. C. Susceptibilities of the transfected parasite lines and the corresponding parental strains to different quinolines. The IC_50_ values to chloroquine (red), quinine (blue), and quinidine (green) were determined in parallel assays for each strain and are shown as the mean ± SEM of at least 8 biological replicates. The corresponding IC_50_ values for the transfectant (open symbol) and the respective parental strain (filled symbol) are plotted in the same line. Statistically different IC_50_ values between transfectant and parental strain are indicated by an asterisk (P<0.01). The relevant genetic markers of the strains are listed in Table 1.

The large size of the *pfut* gene and the position of the key mutations precluded us from using allelic exchange transfection strategies to validate the function of this gene in conferring altered quinine and quinidine responses. We, therefore, pursued an alternative strategy by overexpressing the HECT domain of the *pfut* gene in genetically different *P. falciparum* strains. We reasoned that the polymorphisms in PfUT might affect the activity or substrate specificity of this enzyme and that overexpressing the HECT domain in the cytoplasm might create a dominant negative or positive quinine and quinidine response phenotype in a manner depend on the genetic profile of the recipient strain with regard to *pfcrt*, *pfut*, and the two chromosome 13 QTLs. This approach was inspired by studies conducted in other systems revealing that isolated HECT domains can be enzymatically active [48]. To test this strategy we selected the two parental lines HB3 and Dd2 and five progeny (GC03, CH3-116, TC05, D43, and 7C111), displaying different permutations of the relevant loci (Table 1), and transfected them with a vector expressing a minimal HECT domain fused to GFP. The minimal HECT domain (from amino acids 3652 to 3875) contained all predicted E2 interaction sites and the catalytic cleft including the catalytically active cysteine at position 3860, but lacked parts of the N- and C-terminal lobes that can enhance catalytic activity, as shown in other systems [49]. Cytoplasmic expression of the fusion protein was confirmed by fluorescence microscopy (Figure 9A). The transfected strains maintained the vector at comparable copy numbers per haploid genome of approximately 30, with no significant differences between the transfectants (Figure 9A).

Two of the transfected strains, namely 7C111^UT^ and TC05^UT^, revealed a significant reduction in intracellular quinine accumulation and an almost doubling of the quinine and quinidine IC_50_ values, compared with the corresponding parental stains (P<0.01, Figures 9B and C). These strains harbor, as genomic copies, the *pfut* locus from HB3 and the *pfcrt* locus and the relevant chromosome 13 QTLs from Dd2 (Table 1). A third strain, namely D43^UT^, revealed only a significant reduction in the amount of quinine accumulation (P<0.01, Figure 9B), but no differences in quinine and quinidine resistance (Figure 9C). Although D43^UT^ contains the genomic HB3 *pfut* locus and the genomic Dd2 *pfcrt* locus, it lacks the chromosome 13 QTLs from Dd2 (Table 1) that, as already observed in the QTL analyses and in the *pfcrt* transfectants, contribute to reduced quinine and quindine susceptibility. Overexpression of the HECT domain in HB3, CH3-116, or GC03, which all carry the *pfcrt* locus from HB3 (but different alleles of the *pfut* locus and the chromosome 13 QTLs), had no significant effect on quinine accumulation ratios or quinine and quinidine IC_50_ values, nor was there any effect in Dd2 (Figure 9B and C). The chloroquine responses were unaffected by overexpression of the PfUT HECT domain (Figure 9B and C). Thus, cytoplasmic overexpression of the PfUT HECT domain affected quinine and quinidine response parameters, but only in predisposed genetic backgrounds, with changes in intracellular drug accumulation depending on the presence of the *pfcrt* locus from Dd2 and changes in IC_50_ values depending on the additional presence of the Dd2 chromosome 13 QTLs.

### PfUT functions as a ubiquitin ligase

To verify the enzymatic activity of the HECT domain/GFP fusion protein, we isolated the 110 kDa protein from the transfected Dd2 strain by affinity chromatography using an antibody against GFP. We subsequently tested the enriched protein in *in vitro* ubiqutination assays reconstituted with commercially available human recombinant components including ubiquitin, the E1 activating enzyme UBA, and the E2 conjugating enzymes UBCH5a or UBCH13. The two E2 conjugating enzymes were chosen because they were found to be suitable partners for a *P. falciparum* RING E3 ubiquitin ligase in previous in vitro studies [50]. The enzymatic reactions were performed in a buffer containing an ATP regeneration system and examined by Western analysis.

**Figure 10:**
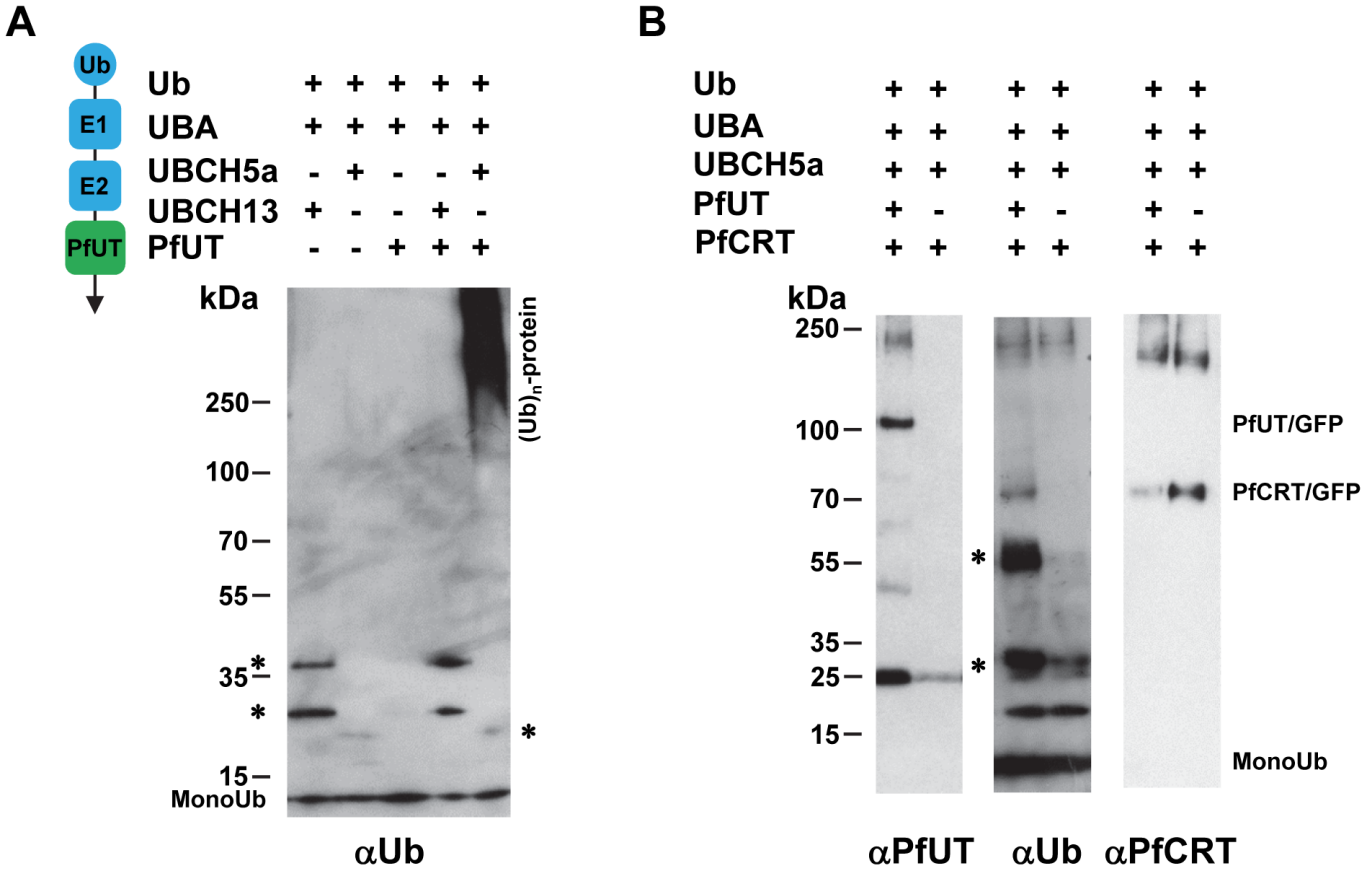
Biochemical characterization of the PfUT HECT domain. A. The PfUT HECT domain/GFP fusion protein catalyzes self polyubiquitination. The PfUT HECT domain/GFP fusion protein was isolated from the corresponding transfected Dd2 line and the catalytic activity of the PfUT HECT domain/GFP fusion protein was tested in an in vitro assay reconstituted with the components indicated. The left scheme indicates the origin of the components and their function. The human components ubiquitin (Ub), E1 activating enzyme (E1; UBA), and the E2 conjugating enzymes (E2; UBCH5a or UBCH13) are highlighted in blue. The PfUT HECT domain/GFP fusion protein (PfUT) is indicated in green. The reactions were examined by Western analysis using SDS PAGE on a 4 -12% gradient gel under non-reducing conditions and an antiserum specific to ubiquitin (αUb, dilution 1∶2000). The asterisks mark ubiquitin intermediate adducts generated by UBCH13 and UBCH5a. High molecular weight ubiquitinated products are indicated. A molecular weight marker is indicated in kDa. A representative example of at least three biological replicates is shown. The supplementary Figure S6A shows an independent biological replicate and supplementary Figure S6B shows absence of enzymatic activity when parasite purified GFP was used in the assay instead of the PfUT HECT domain/GFP fusion. B. The PfUT HECT domain/GFP fusion protein catalyzes ubiquitination of substrate proteins. *In vitro* ubiquitination assays were reconstituted using the components indicated, including a PfCRT/GFP fusion protein isolated from the corresponding transfected Dd2 line. The reactions were examined by Western analysis using SDS PAGE on a 4–12% gradient gel under non-reducing conditions and antisera specific to the PfUT HECT domain (αPfUT, dilution 1∶5000), ubiquitin (αUb, dilution 1∶2000), and PfCRT (αPfCRT, dilution 1∶1000). In addition to PfCRT/GFP, the immunoglobulin heavy (53 kDa plus ubiquitin) and light chains (25 kDa plus ubiquitin) (present in the reaction because the conditions required to elute PfCRT/GFP also eluted immunoglobulins from the column) were ubiqutinated and are indicated by asterisks.

An antibody against ubiquitin detected high molecular weight ubiquitinated products only in the reaction containing the PfUT HECT domain and that only when reconstituted with the E2 conjugating enzyme UBCH5a (Figure 10A; supplementary Figure S6A). Reactions that did not contain the PfUT HECT domain or in which UBCH13 replaced UBCH5a were catalytically inactive (Figure 10A; supplementary Figure S6A). High molecular weight ubiqutinated products in the absence of a substrate protein are characteristic of some HECT ubiquitin ligases and are explained by spontaneous self polyubiquitination of internal lysines, following formation of the thioester adduct [48], [51]. No enzymatic activity was observed when, instead of the HECT domain/GFP fusion protein, GFP only was tested. GFP was isolated from a transgenic Dd2 line following the protocol established for the HECT domain/GFP fusion (supplementary Figure S6B).

Next we added enriched PfCRT/GFP as a putative substrate to the active reaction. PfCRT/GFP was isolated from transfected Dd2 parasites by affinity chromatography using an antiserum against GFP. The antibody against ubiquitin detected the PfCRT/GFP fusion protein and the immunoglobulin heavy (53 kDa) and light chains (25 kDa) (the conditions required to elute PfCRT/GFP from the column also eluted immunoglobulins) but only in reactions containing the PfUT HECT domain and not in the control (Figure 10B). Reprobing the membrane with an antiserum against PfUT confirmed the presence of the PfUT HECT domain/GFP fusion protein of 110 kDa in the enzymatically active reaction, whereas the negative control did not contain the protein (Figure 10B). The PfCRT/GFP fusion protein of 75.6 kDa was present in both reactions (Figure 10B). Apparently, the PfUT HECT domain catalyzed the ubiquitination of the PfCRT/GFP fusion protein and of immunoglobulins. These data suggest that the HECT domain/GFP fusion expressed in the various *P. falciparum* transfectants is enzymatically active.

## Discussion

Although quinine has been used in the treatment of malaria since the 17^th^ century, it remains a fairly effective drug with cure rates generally exceeding 90%. High failure rates of ≥20% have been reported only from Venezuela and Cambodia [52]. There is, however, a general progressive decline in the sensitivity of *P. falciparum* strains to quinine across all malaria endemic areas [52]–[55], which increasingly threatens the clinical efficacy of this important antimalarial drug. The very slow genesis of quinine resistance argues for a highly complex underpinning mechanism, one that out-matches that of the structurally related antimalarial drug chloroquine. In comparison, chloroquine resistance emerged in as few as 12 years after the drug was introduced in the field. It primarily results from multiple mutations in the *pfcrt* gene [56].

A previous study has mapped altered quinine responsiveness in the HB3 x Dd2 cross as a Mendelian trait to segments on chromosomes 5, 7 and 13, containing the polymorphic genes *pfcrt*, *pfmdr1*, and *pfnhe*, respectively [15]. Our re-analysis of quinine responses in the HB3 x Dd2 cross has revealed a more refined picture. We confirmed the association with *pfcrt* and *pfmdr1* and, in addition, identified a novel candidate gene, termed MAL7P1.19 (or *pfut*) encoding a HECT ubiquitin-protein ligase [35]. Our data further shed new light on the role of the *pfnhe* containing segment on chromosome 13. This segment shows an interesting dichotomy. It contributes to reduced quinine and quinidine susceptibility, but not to differential drug accumulation. This is a surprising finding. Previous studies have suggested that the contributing gene within the chromosome 13 segment is *pfnhe* [15] and that polymorphisms in, and/or altered expression levels of, *pfnhe* might affect intracellular pH homeostasis [19], which in turn might impact on drug partitioning, particularly if the drug has acidotropic properties as do quinine and quinidine. However, we did not find such an effect. Differential intracellular quinine and quinidine accumulation was independent of the *pfnhe* expression level, as shown by investigating mutants with partially knocked-down *pfnhe* expression, and it was independent of polymorphisms in PfNHE even though PfNHE of HB3 and Dd2 differ by several amino acid substitutions and length polymorphisms. This raises the possibility of factors other than *pfnhe* contributing to altered quinine and quinidine susceptibility [57], [58]. This possibility is further nurtured by *pfnhe* residing in a valley of what might be a doublet QTL, defined by the markers VAPA and C13M73, although the evidence for a doublet QTL is only circumspect. The defining property of the chromosome 13 segment, that it decreased quinine and quinidine susceptibility while having no noticeable effect on quinine and quinidine accumulation (Figures 1 and 2), might suggest that it encodes factors that are targeted by quinine and quinidine.

The *pfut* gene remained unnoticed in the initial study by Ferdig et al. (2004) [15], probably because the relevant LOD scores (B5M12 linkage group), derived from cell proliferation assays, only just reached significance in the analysis of the quinine IC_50_ and IC_90_ values. In contrast, the LOD scores derived from our accumulation studies were four to five magnitudes higher, providing sufficient statistical power to link the *pfut* locus with quinine response variants. Furthermore, unlike Ferdig et al. (2004), we have based our genetic linkage analysis on not just one assay but on two independent assays (the standard cell proliferation assay and the drug accumulation assay) and have independently confirmed the results with the related drug quinidine. Thereby our results achieved a high degree of confidence.

Both *pfut* and *pfcrt* reside on chromosome 7 in genetic linkage groups that are 14.4 cM or, alternatively, 97 kb apart (supplementary Table S2). Although both loci are physically linked, they are, in genetic terms, sufficiently apart to unequivocally separate them from one another in the QTL analyses of the HB3 x Dd2 cross. The reported average recombination distance of ∼15 kb per cM in this cross allows loci to be mapped within segments of 15–50 kb [37]. Further aiding in the parsing of the *pfut* and *pfcrt* loci, there is a saturating number of independent recombination events in the part of chromosome 7 surrounding *pfcrt* because of previous efforts to resolve the chloroquine resistance locus, which eventually led to the discovery of *pfcrt* [37], [56]. Of the 34 progeny, five carry distinct recombination events in the interjacent chromosomal domain between *pfut* and *pfcrt*, allowing us to parse both loci with high statistical power (P = 0.0008).

In comparison, in another genetic cross, between the *P. falciparum* strains 7G8 and GB4, none of the progeny harbor a recombination event between the *pfut* and *pfcrt* loci [38]. Accordingly, both markers form a single genetic linkage group in this cross. This explains why a recent study associated altered quinine and quinidine responses in the 7G8 x GB4 cross with a single QTL on chromosome 7, and not two, despite 7G8 and GB4 carrying distinct *pfut* and *pfcrt* haplotypes [42].

The association studies on polymorphic markers in 50 field isolates and strains revealed that *pfut* is not part of the chromosomal domain of restricted genetic diversity that surrounds *pfcrt* in chloroquine resistant *P. falciparum* strains [44]–[46]. There are at least twelve polymorphic genes immediately downstream of *pfut* towards *pfcrt*, none of which were associated with mutant *pfcrt*. In comparison, RAMA, which is one of the genes closest to *pfcrt* in the B5M12 locus, seems to be part of the low diversity *pfcrt* linkage disequilibrium block. RAMA and the genes following it on the chromosome in the direction towards *pfcrt* are all highly associated with mutant *pfcrt*, suggesting that they have been passed on from one parasite generation to the next as part of a conserved chromosomal domain.

*pfut* seems to contribute to quinine and quinidine response variations only when paired with other traits, particularly with *pfcrt* and a segment on chromosome 13. A possible functional association between *pfut* and *pfcrt* is supported by the drug accumulation assays performed with the progeny of the HB3 x Dd2 cross (Figures 1B and 2B) and with the pfcrt and PfUT HECT domain transfectants (Figures 4D; Figure 9B) where mutant *pfcrt* could only bring about a significant reduction in drug accumulation levels when partnered with the variant form of *pfut* or an overexpressed PfUT HECT domain. However, the *pfcrt* and *pfut* associated reduction in intracellular drug levels did not necessarily result in reduced susceptibility as defined by an IC_50_ or IC_90_ value. In two of the five genetic backgrounds investigated there was a reciprocal correlation between drug accumulation and resistance, but in three other genetic backgrounds the combined effect of the *pfcrt* and *pfut* genes was neutral or even resulted in increased drug sensitivity (compare transfectants and corresponding parental strains in Figures 4 and 9). The explanation offered by the QTL analysis is that these strains lack the chromosome 13 QTL(s) that, as discussed above, might encode targets of quinine and quinidine. Indeed, all the transfectants in which *pfcrt* and *pfut* conferred increased quinine and quinidine resistance also harbored the relevant chromosome 13 segment from Dd2. Those transfectants that lacked the Dd2 chromosome 13 segment revealed no changes in susceptibility or became even more susceptible, despite the expression of the variant form of *pfcrt* and the variant or overexpressed form of *pfut*. The absence of a reciprocal correlation between PfCRT-mediated drug efflux from the digestive vacuole, as defined by a reduced net intracellular drug accumulation ratio, and drug susceptibility has recently also been noted and is explained by the altered drug flux enhancing the encounter of the drug with targets outside the digestive vacuole [42].

The complex, multifactorial nature of quinine resistance explains allelic exchange experiments in which polymorphic *pfcrt* alleles were introduced into the genetic background of the *P. falciparum* clone GC03 (a progeny from the HB3 x Dd2 cross) [27]. These mutants were highly susceptible to quinine and quinidine. We explain this puzzling result by the fact that these mutants carry the wild-type HB3-like *pfut* gene and the wild type chromosome 13 QTLs, thus lacking essential factors required for reduced quinine responsiveness. Similarly, the lack of a correlation between mutant *pfcrt* and quinine resistance in some field isolates may be because these strains are wild-type in the relevant other loci.

Unexpectedly, low quinine responding strains (as defined by an IC_50_ value >100 nM) from Latin America and Southeast Asia and also the two African strains investigated share a conserved set of polymorphisms within PfUT (Figure 7B), despite distinct regional histories of drug use and drug selection, as evidenced by their different *pfcrt* variants [38], [42]. This conservation has to be considered in the context of the overall highly polymorphic nature of *pfut*. We noted at least 19 non-synonymous mutations and several length polymorphisms in this gene between HB3 and Dd2. Apparently, the conserved set of polymorphisms within *pfut* has independently emerged in Latin America and in Southeast Asia, suggesting that *pfut* is under a strong positive selective pressure [23], possibly from the use of quinine, although previous genome wide association studies failed to establish a link between *pfut* and quinine response variations [23], [24]. The conserved polymorphic residues reside in a domain of PfUT that reveals a high degree of phylogenetic diversity among orthologs in other Plasmodia.

Whether *pfut* contributes to drug response variations other than quinine and quinidine is still under investigation. *pfut* does not seem to contribute to resistance to amodiaquine or its active metabolite desethyl amodiaquine [38], [42], compounds that shares the quinolone scaffold with quinine and chloroquine, or to artemisinin [59]. There is, however, evidence implicating the *pfut*-carrying B5M12 locus in altered responses to diphemanil and, possibly, chloroquine [40], [60]. An effect of the B5M12 locus on altered chloroquine responsiveness in the HB3 x Dd2 cross, however, is controversial and only supported by the QTL analysis on the chloroquine IC_50_ values reported by Patel et al. (2010), but not by the Sa et al. (2009) or our own data sets [38], [40], [42].

How PfUT affects quinine responses is currently unclear. PfUT localizes to the parasite's ER/Golgi complex where it seems to form high molecular weight complexes of >1 MDa, as shown in a native blue gel, possibly by associating with factors of the proteasome [61]. However, PfUT does not seem to be part of the ER-associated degradation pathway that mediates proteolysis of misfolded proteins [62]. PfUT shares sequence homologies with the *Saccharomyces cerevisiae* UFD4 HECT ubiquitin-protein ligase [35]. UFD4 was initially identified as a component of the ubiquitin fusion degradation pathway that recognizes an N-terminal ubiquitin moiety and which targets these ubiquitin fusion proteins for polyubiquitination and degradation [63]. Later it was found that UFD4 is also involved in the Arg/N-end rule pathway, by augmenting the processivity of polyubiquitination of Arg/N-end rule substrates [36]. Thus, PfUT might play a role in proteasome-mediated degradation. A yeast-two-hybrid screen has revealed an interaction of PfUT with several proteins [61] that might be substrates of PfUT. This includes the chloroquine resistance marker protein (CRMP), a putative nuclear target of chloroquine [64].

There are several examples in the literature where mutations in an ubiquitin ligase or a deubiquitination enzyme affect drug susceptibilities, frequently by regulating the stability of a resistance-mediating drug transporter [65]–[67]. An example from a malaria parasite is a deubiquitination enzyme that is associated with increased resistance to artemisinin and chloroquine in *Plasmodium chabaudi* [68]. Since both PfUT and PfCRT spatially and temporally overlap, as PfCRT traffics to the parasite's digestive vacuole via the parasite's ER/Golgi complex [69], it is tempting to speculate that PfUT acts on PfCRT and that mutational changes in, or overexpression of, PfUT affect PfCRT's trafficking, final conformation, longevity, and/or activity. Although the isolated PfUT HECT domain ubiquitinated a PfCRT/GFP fusion protein in an *in vitro* reconstituted ubiquitination assay (Figure 10B), further experiments are needed to validate this finding and demonstrate ubiquitination of native PfCRT in the parasite. A previous proteomic analysis of PfCRT purified from the parasite revealed phosphorylation at serine 33, serine 411, and threonine 416, with the last posttranslational modification being a defining signal for trafficking of PfCRT to the digestive vacuole [69]. Ubiquitination of PfCRT was not detected in this study, possible because the N-terminal domain of PfCRT, which is the likely site of action of PfUT, was not covered in this study. In general, coverage was poor in regions other than the C-terminal cytoplasmic domain. In summary, our study presents several independent lines of evidence suggestive of *pfut* playing a role in altered quinine and quinidine responses in certain genetic backgrounds. Definitive proof, however, would await allelic exchange experiments and extended surveys of clinical isolates to validate the association of polymorphisms in *pfut* with reduced quinine susceptibility.

## Materials and Methods

### *P. falciparum* strains and cultures

*P. falciparum* was cultured as previously described [70] and synchronized using the sorbitol method [71]. The F1 progeny from the HB3 x Dd2 cross have been described [37] and were obtained from MR4. The identity of all progeny was verified by PCR using eight highly polymorphic microsatellite markers as described [42].

### Transfection and live cell imaging

The transfection vector expressing *pfcrt* tagged in frame with the coding sequence of the green fluorescence protein has recently been described [69]. Briefly, the *pfcrt* coding sequence from the chloroquine resistant *P. falciparum* strain Dd2 or the wild type sequence from the chloroquine sensitive strain HB3 was cloned into the *Xho*I and *Avr*II restricted pARL1a^+^ transfection vector [72] in a manner that allowed *pfcrt* expression to be controlled by its own promoter. The GFP coding sequences was subsequently cloned into *Avr*II and *Xma*I restricted pARL1a^+^ vector containing the *pfcrt* coding sequence to create a C terminal PfCRT/GFP fusion. The catalytic domain of PfUT encompassing the amino acids 3653 to 3877 were cloned in frame with the coding sequences of the conditional aggregation domain [73] and GFP into *Xho*I and *Avr*II restricted pARL1a^+^. Parasites were transfected using 100 µg plasmid DNA, and transfectants were selected using 5 nM WR99210, as previously described [74]. Transfectants were detected in blood smears 14–21 days post transfection. Transfectants were grown in the presence of 5 nM WR99210 until two days before analysis when the drug pressure was removed to avoid interference of WR99210 with the drug accumulation assay or the growth inhibition assay. Live cell imaging of *P. falciparum*-infected erythrocytes were performed as described [69].

### Radio-chemicals

Radiolabeled quinoline antimalarial drugs were obtained from the following vendors: [^3^H]-chloroquine (18.8 Ci/mmol), GE Healthcare; [^3^H]-quinine (20 Ci/mmol) and [^3^H]-quinidine (20 Ci/mmol), American Radiolabeled Chemicals, Inc.

### Drug accumulation assay

The drug accumulation assay has been fully described [21], [75]. Briefly, *P. falciparum*-infected erythrocytes were purified using a strong magnet (VarioMACS, Miltenyi Biotec), as described [75]. This yielded a purity of trophozoite-infected erythrocytes of 95–100% as determined by light microscopic examination of Giemsa-stained blood smears. The cells were resuspended in prewarmed RPMI 1640 containing 11 mM glucose, 25 mM HEPES, and 2 mM glutamine (pH 7.3 at 37°C) at an haematocrit of 25000 cells/µl. The haematocrit was determined using an automated cell counter (Z1-Coulter Particle Counter, Beckman Coulter Inc.). Cells were then incubated at 37°C in the presence of 40 nM of the respective drug and the amount of label accumulated over time was monitored. The cellular drug accumulation ratio was determined as described [75]. Throughout the study, trophozoite-infected erythrocytes (20–28 hrs post invasion) were examined. The accumulation ratios to quinine, quinidine and chloroquine were determined in parallel assays.

### Quinine, quinidine, and chloroquine half maximal inhibitory concentrations

Cell proliferation assays in the presence of different concentrations of chloroquine, quinine, and quinidine, were performed as described [15]. For the *pfcrt* and *pfut* HECT domain transfectant parasite lines, the IC_50_ values to these drugs were determined in parallel assays over a period of four months. The quinine IC_90_ values in the presence and absence of 0.89 µM verapamil have been described for the HB3 x Dd2 cross [15]. The corresponding quinine IC_50_ values for the HB3 x Dd2 cross were derived from these original data and are compiled in supplementary Table S1. The quinine and chloroquine IC_50_ values for the 50 field isolates and laboratory strains were taken from Mu et al. (2003) [43].

### QTL analysis

QTL analysis was performed as described [76] and validated using R/QTL. The percent contribution of individual QTLs to the total variance in a response parameter was calculated using R/QTL. The genetic maps have been published [38]. The QTL analysis for the field strains was performed in a similar fashion. The quinine susceptibility of a strain was correlated with the presence or absence of a certain polymorphism, yielding the Pearson coefficient and from this, the probability, as the P value. The appropriate LOD score for a locus was computed as the logarithm of the probability P at that locus divided by the mean of the P values taken over all the loci. LOD scores above 2 (a P value one-hundredth that of the mean) were deemed significant.

### Statistical analysis

Data were analysed using one or two way ANOVA test (Holm-Sidak test), or the Student's t-test, where appropriate. Statistical calculations were done using SigmaPlot 12.5.

### Comparative genome analysis

Appropriate regions of the HB3 and Dd2 genomes were downloaded from the Broad Institute MIT database and analyzed using the BLAST algorithm to identify putative polymorphisms. Novel SNPs, indels, and microsatellite markers have been reported to Genbank.

### Indirect immunofluorescence

Rabbit and mouse antisera were generated against the N-terminal (residues 473 to 712), the C-terminal domain of PfUT (residues 3654 to 3875) and against two combined peptides (DYNIKEDDESGSSN and LDDGVRPEKRKT). Immunofluorescence was carried out on magnet purified trophozoites [42] fixed with 4% EM-grade paraformaldehyde (EMS) and 0.0075% EM-grade glutaraldehyde (Merck) in PBS for 30 min [77]. Primary antisera were diluted as follows: rabbit α-PfBiP 1∶1000; rat α-PfERD2 1∶500; rabbit N-terminal PfUT 1∶3000; rabbit C-terminal PfUT 1∶3000; mouse N-terminal PfUT 1∶2000. Corresponding secondary antibodies were used at a dilution of 1∶1000. Slides were viewed using a LSM510 laser scanning confocal microscope (Carl Zeiss).

### Immuno electron microscopy

Immuno electron microscopy was performed as described [78], using the rabbit antisera against the N-terminal domain of PfUT (1∶100) coupled to 10 nm protein A colloidal gold.

### Western analysis

For Western analysis, the following antisera were used: guinea pig anti-PfCRT (dilution 1∶1000) [79] and as secondary antibody donkey anti guinea pig antibodies conjugated with horseradish peroxidase (POD) (1∶10000, Dianova); monoclonal mouse anti-GFP (dilution 1∶1000, Roche Diagnostics) and as secondary antibody goat anti mouse POD (1∶10000, Dianova); rabbit anti the C-terminal domain of PfUT (residues 3652 to 3875) (1∶5000) and as secondary antibody goat anti rabbit POD (1∶10000, Dianova); monoclonal-mouse anti-ubiquitin (1∶2000, Santa Cruz Biotechnology) and as secondary antibody goat anti mouse POD (1∶10000, Dianova). POD activity was detected using the BM Chemiluminescence Blotting Substrate Kit (Roche Diagnostics).

For the analysis of native proteins and protein complexes, magnet purified trophozoites were saponin-lysed (0.07% in PBS), washed two times in PBS, and subsequently solubilized using increasing concentrations of Triton X-100 ranging from 0.125% to 1% with mixing at 4°C for 30 min. All solutions were supplemented with protease inhibitors (1 mM PMSF, 50 µg/ml aprotinin, 20 µg/ml leupeptin). Insoluble material was pelleted (14,000 g for 30 min at 4°C), and supernatant fractions were collected. Samples were subsequently analyzed using a native blue gel as described [80].

For the analysis of membrane proteins, a Triton X-114 phase separation protocol was used [81]. Briefly, saponin-lysed magnet purified trophozoites were incubated in a Triton X-114 buffer (1% Triton X-114, 150 mM NaCl, 10 mM Tris-Cl pH 7.4) for 3 min at 30°C before centrifuged at 300 g. The supernatant was removed and subject to a second round of extraction (0.5% final concentration of Triton X-114). The lower detergent phase was analyzed by SDS-PAGE on a 3–8% gradient gel followed by Western analysis.

### Quantification of vector copy number

The DNA from approximately 2×10^8^ parasites was isolated using the DNeasy Blood & Tissue Kit from Qiagen. Copies of pARL plasmids were subsequently determinate by quantitative real time PCR as described [82], using primers to the bla gene. The reaction was performed using the LigthCycler DNA Master SYBR Green I reaction mix and the Biorad CFX96 Real-Time System. In parallel reactions, the amount of genomic DNA was determined by quantitative real time PCR, using primers to the *P. falciparum* β-tubulin gene, as described [83]. The normalized number of plasmid copies per haploid genome was then obtained. The high copy numbers reported in this study are consistent with episomal maintenance of the transfection vector, although occasional genomic integration cannot be excluded.

### *In vitro* reconstituted ubiquitination assay

The GFP fusion proteins PfCRT/GFP and PfUT HECT domain/GFP were isolated from the respective transfectant Dd2 lines as described [69]. Briefly, following synchronisation with 5% sorbitol [71], infected erythrocytes at the trophozoite stage were purified from 600 ml parasite culture (5% haematocrit and 8% parasitemia) using the Super Macs magnet (Miltenyi Biotech) and the D column. Infected erythrocytes were subsequently treated with 0.07% saponin in PBS. Immunoprecipitation was performed as described [73]. Briefly, proteins were extracted using the RIPA buffer for PfCRT (1% NP-40, 1% sodium deoxycholate, 0.1% SDS, 150 mM NaCl, 10 mM Na-Phosphate buffer pH 7, 1 mM EDTA) and an TNE buffer for PfUT (20 mM Tris-HCl pH 7.5, 137 mM NaCl, 1% NP40, 2 mM EDTA, plus protease inhibitors: leupeptin 20 µg/ml, aprotinin 50 µg/ml, PMSF 1 mM). The lysate was then diluted with 9 volumes of NETT buffer (10 mM Na-Phosphate buffer pH 7, 150 mM NaCl, 0.1% NP-40, and 1 mM EDTA). Prior to immuno-precipitation, lysates were cleared using goat IgGs. For immunoprecipitation, the ProFound™ Co-Immunoprecipitation Kit (Pierce) was used according to the manufacturer's instructions, using goat anti-GFP antiserum (Rockland). Immuno-precipitated material was washed in buffers with increasing NaCl concentrations ranging from 250 to 350 mM and one additional wash with 50 mM Tris HCl pH 7.5. All buffers used were supplemented with a cocktail of protease and phosphatase inhibitors (50 µg/ml aprotinin, 20 µg/ml leupeptin, 1 mM PMSF).

*In vitro* ubiqitination assays were performed as described [50] and examined by Western analysis. The following reagents purchased from Boston Biochem were used in the ubiquitination assay: 70 µM human recombinant ubiquitin, 200 nM human recombinant ubiquitin activating enzyme UBA, 5 µM human recombinant ubiquitin conjugating enzyme UbcH5a or UbcH13, and 1 x energy regeneration solution. The reactions were size-fractionated by non-reducing SDS PAGE on a 4–12% gradient gel, transferred to polyvinylidene difluoride membranes, and analyzed using the antisera indicated (see also above).

## Supporting Information

Figure S1 Time courses of quinine and quinidine accumulation. A. Time courses of net intracellular quinine accumulation by the *P. falciparum* clones HB3 (filled circles) and Dd2 (open circles) from an external concentration of 40 nM. The amount of intracellular drug accumulated is given as the ratio of the intracellular over the extracellular drug concentration. B. Time courses of net intracellular quinidine accumulation from an external concentration of 40 nM. The mean ± SEM of six independent biological replicates is shown. QN, quinine; QD, quinidine.
(PDF)

Figure S2 Linkage analyses on quinine IC_50_ values and 25 min quinine accumulation ratios in the HB3 x Dd2 cross. A. The net intracellular quinine accumulation ratios (QN_in_/QN_out_) were determined in the F1 progeny from the genetic cross between HB3 and Dd2 and in the two parental strains after 25 min of incubation (steady state phase). The means ± SEM of at least 8 independent determinations are shown. The quinine IC_50_ values for the progeny and the two parental clones were derived by reanalysis of the quinine IC_90_ values published by Ferdig et al. (2004) [15]. Progeny containing the wild-type *pfcrt* of HB3 and the polymorphic *pfcrt* of Dd2 are indicated. B. QTL analyses on the net intracellular quinine accumulation ratios (black line) and the quinine IC_50_ values. The logarithm of odds (LOD) scores from the primary scans are shown as a function of genome location. The *pfcrt* and B5M12 loci on chromosome 7 and the bifurcated peak on chromosome 13 are indicated. The dotted lines represent the confidence line with p<0.01. C. Enlarged display of the bifurcated peak on chromosome 7.
(PDF)

Figure S3 Linkage analyses on 25 min quinidine accumulation ratios in the HB3 x Dd2 cross. A. The net intracellular quinidine accumulation ratios (QD_in_/QD_out_) were determined in the F1 progeny from the genetic cross between HB3 and Dd2 and in the two parental strains after 25 min of incubation (steady state phase). The means ± SEM of at least 8 independent determinations are shown. B. QTL analyses on the net intracellular quinidine accumulation ratios (black line) and the quinidine IC_50_ values are shown. Relevant genetic markers are indicated. C. Enlarged display of the bifurcated peak on chromosome 7.
(PDF)

Figure S4 Schematic illustration of the B5M12 and *pfcrt* locus on chromosome 7, including relevant genetic markers and chromosomal position (in Mbp). Genes encoded within the B5M12 locus are indicated. The MAL7P1.19 encoding a HECT ubiquitin-protein ligase is highlighted.
(PDF)

Figure S5 Effect of an episomally expressed wild type *pfcrt* allele on chloroquine (A) and quinine accumulation (B). Chloroquine and quinine accumulation levels were determined at the 20 min time point in transfected parasite lines and the corresponding parental strains. The means ± SEM of at least 10 independent determinations are shown. *, P<0.001. The genetic background of the parasite lines with regard to the genomic copy of *pfcrt* and the B5M12 locus are: HB3 and GCO3, *pfcrt* ^HB3^ B5M12^HB3^; CH3-116, *pfcrt* ^HB3^ B5M12^Dd2^.
(PDF)

Figure S6 Parasite purified PfUT HECT domain/GFP fusion, but not GFP, catalyzes self polyubiquitination. A. The PfUT HECT domain/GFP fusion protein catalyzes self polyubiquitination. An independent biological replicate to the data presented in Figure 10A are shown. The PfUT HECT domain/GFP fusion protein was isolated from the corresponding transfected Dd2 line and the catalytic activity of the PfUT HECT domain/GFP fusion protein was tested in an in vitro assay reconstituted with the components indicated. The left scheme indicates the origin of the components and their function. The human components ubiquitin (Ub), E1 activating enzyme (E1; UBA), and the E2 conjugating enzymes (E2; UBCH5a or UBCH13) are highlighted in blue. The PfUT HECT domain/GFP fusion protein (PfUT) is indicated in green. The reactions were examined by Western analysis using SDS PAGE on a 4–12% gradient gel under non-reducing conditions and an antiserum specific to ubiquitin (αUb, dilution 1∶2000). The asterisk marks ubiquitin intermediate adducts generated by UBCH13 and UBCH5a. High molecular weight ubiquitinated products are indicated. A molecular weight marker is indicated in kDa. B. A parasite purified GFP is enzymatically inactive. Same experiment as described above, but this time parasite purified GFP was used. GFP was purified from a genetically engineered Dd2 line following the protocol established for the purification of PfUT HECT domain/GFP fusion.
(PDF)

Table S1 The enclosed excel file contains the IC_50_ values and the accumulation ratios of the progeny from the HB3 x Dd2 cross.
(XLS)

Table S2 Genetic map of the chromosome 7 segment containing the B5M12 locus and the *pfcrt* locus.
(XLS)

Table S3 Primer pairs defining novel genetic markers within the B5M12 locus for the analysis of the progeny from the HB3 x Dd2 cross.
(XLS)

Table S4 Compilation of relevant quantitative trait loci, LOD scores, and corresponding correlation coefficients. Only QTLs that rose above the confidence line of p<0.01 were considered. The chromosomal position of the markers in centi Morgan (cM) is indicated. A positive correlation coefficient (κ) indicates that the marker from Dd2 contributes to a decrease in drug susceptibility and an increase in drug accumulation. A negative correlation coefficient indicates that the marker from Dd2 contributes to an increase in drug susceptibility and a reduction in drug accumulation. QTLs identified in primary scans are highlighted in bold. QN, quinine; and QD, quinidine.
(PDF)

Table S5 Compilation of polymorphisms identified in the B5M12 locus and flanking regions in 50 field isolates and laboratory strains.
(XLS)
