# Supplementary figures and images for: A HECT Ubiquitin-Protein Ligase as a Novel Candidate Gene for Altered Quinine and Quinidine Responses in Plasmodium falciparum
Source: PLoS Genet. 2014 May 15;10(5):e1004382. doi: 10.1371/journal.pgen.1004382 (PMC4022464; doi:10.1371/journal.pgen.1004382)

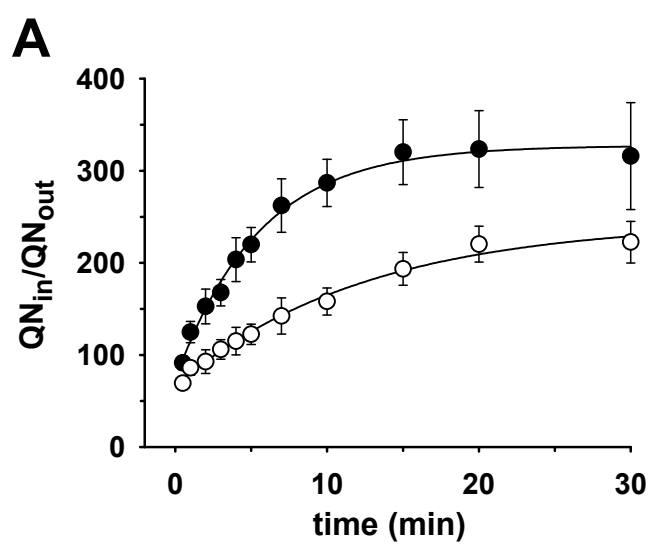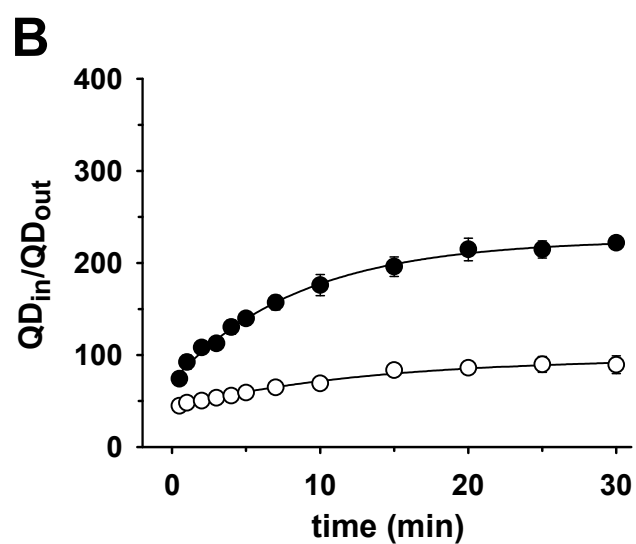

**Figure S1**

Supplement: Figure S1 — Time courses of quinine and quinidine accumulation. A. Time courses of net intracellular quinine accumulation by the P. falciparum clones HB3 (filled circles) and Dd2 (open circles) from an external concentration of 40 nM. The amount of intracellular drug accumulated is given as the ratio of the intracellular over the extracellular drug concentration. B. Time courses of net intracellular quinidine accumulation from an external concentration of 40 nM. The mean ± SEM of six independent biological replicates is shown. QN, quinine; QD, quinidine. (PDF) [file pgen.1004382.s001.pdf]

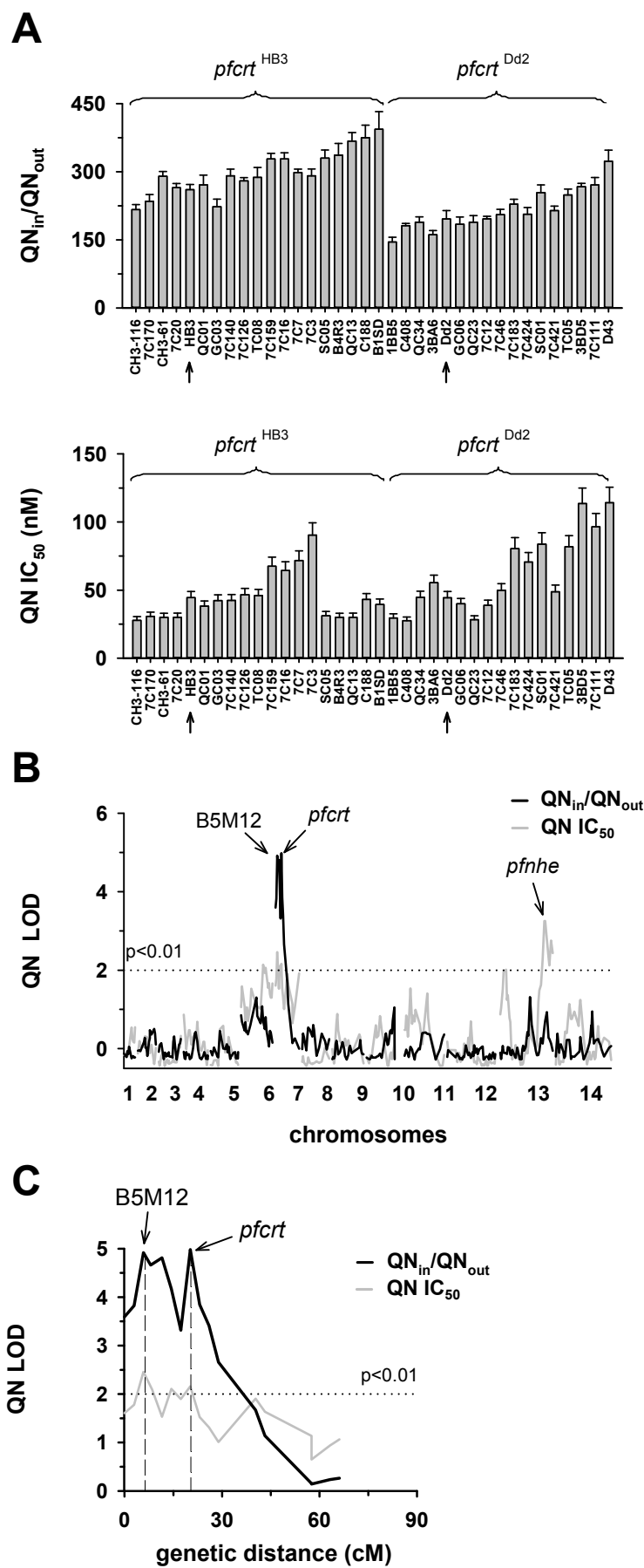

**Figure S2**

Supplement: Figure S2 — Linkage analyses on quinine IC50 values and 25 min quinine accumulation ratios in the HB3 x Dd2 cross. A. The net intracellular quinine accumulation ratios (QNin/QNout) were determined in the F1 progeny from the genetic cross between HB3 and Dd2 and in the two parental strains after 25 min of incubation (steady state phase). The means ± SEM of at least 8 independent determinations are shown. The quinine IC50 values for the progeny and the two parental clones were derived by reanalysis of the quinine IC90 values published by Ferdig et al. (2004) [15]. Progeny containing the wild-type pfcrt of HB3 and the polymorphic pfcrt of Dd2 are indicated. B. QTL analyses on the net intracellular quinine accumulation ratios (black line) and the quinine IC50 values. The logarithm of odds (LOD) scores from the primary scans are shown as a function of genome location. The pfcrt and B5M12 loci on chromosome 7 and the bifurcated peak on chromosome 13 are indicated. The dotted lines represent the confidence line with p<0.01. C. Enlarged display of the bifurcated peak on chromosome 7. (PDF) [file pgen.1004382.s002.pdf]

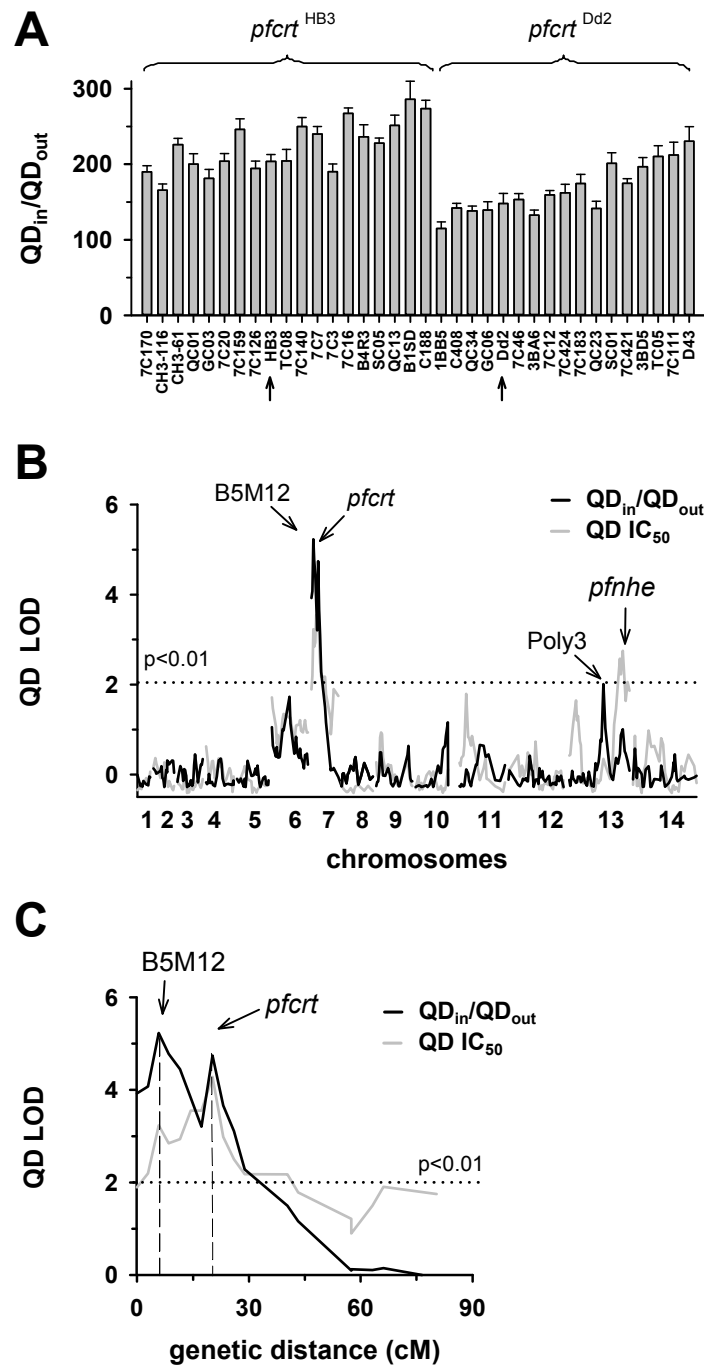

**Figure S3**

Supplement: Figure S3 — Linkage analyses on 25 min quinidine accumulation ratios in the HB3 x Dd2 cross. A. The net intracellular quinidine accumulation ratios (QDin/QDout) were determined in the F1 progeny from the genetic cross between HB3 and Dd2 and in the two parental strains after 25 min of incubation (steady state phase). The means ± SEM of at least 8 independent determinations are shown. B. QTL analyses on the net intracellular quinidine accumulation ratios (black line) and the quinidine IC50 values are shown. Relevant genetic markers are indicated. C. Enlarged display of the bifurcated peak on chromosome 7. (PDF) [file pgen.1004382.s003.pdf]

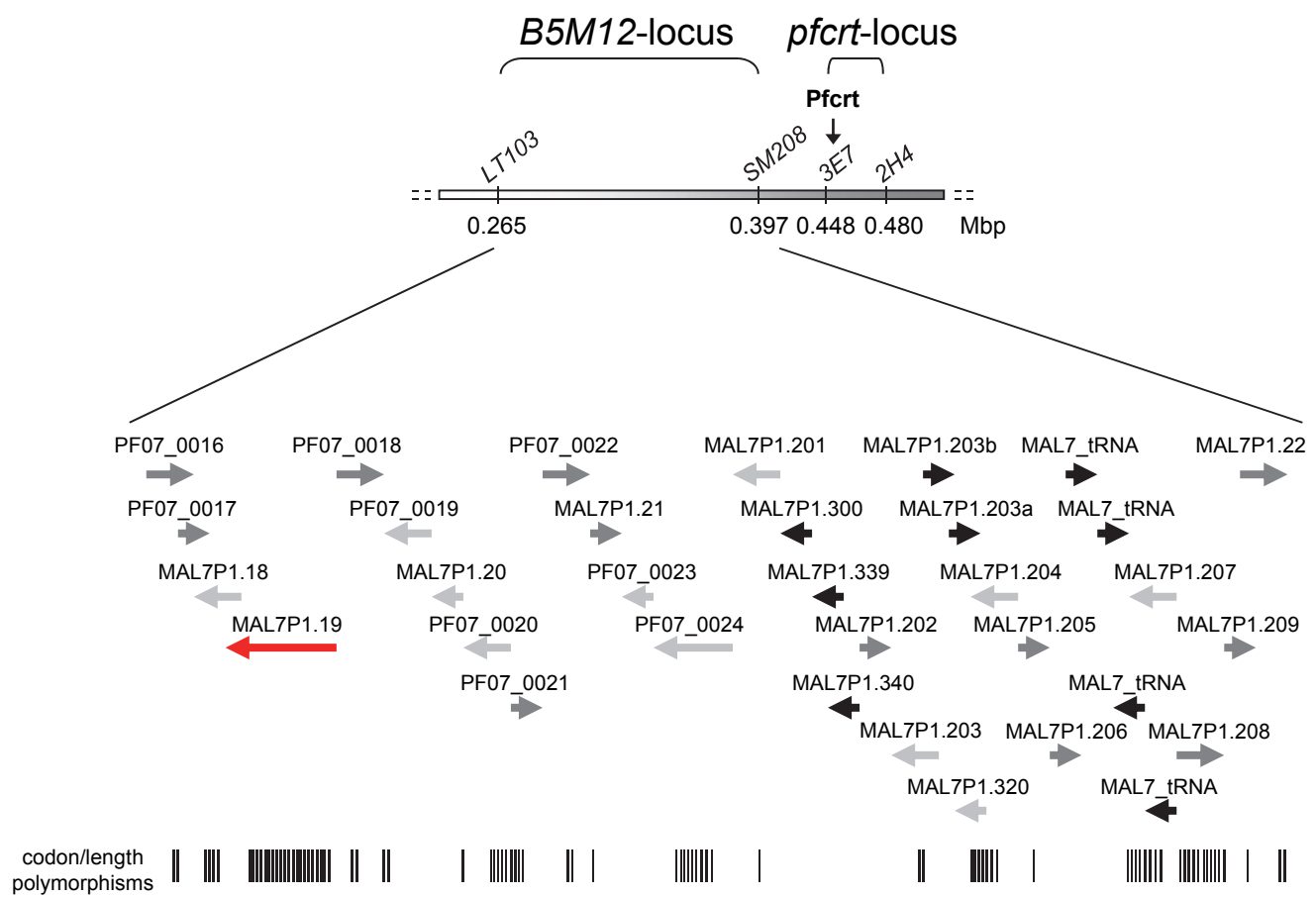

**Figure S4**

Supplement: Figure S4 — Schematic illustration of the B5M12 and pfcrt locus on chromosome 7, including relevant genetic markers and chromosomal position (in Mbp). Genes encoded within the B5M12 locus are indicated. The MAL7P1.19 encoding a HECT ubiquitin-protein ligase is highlighted. (PDF) [file pgen.1004382.s004.pdf]

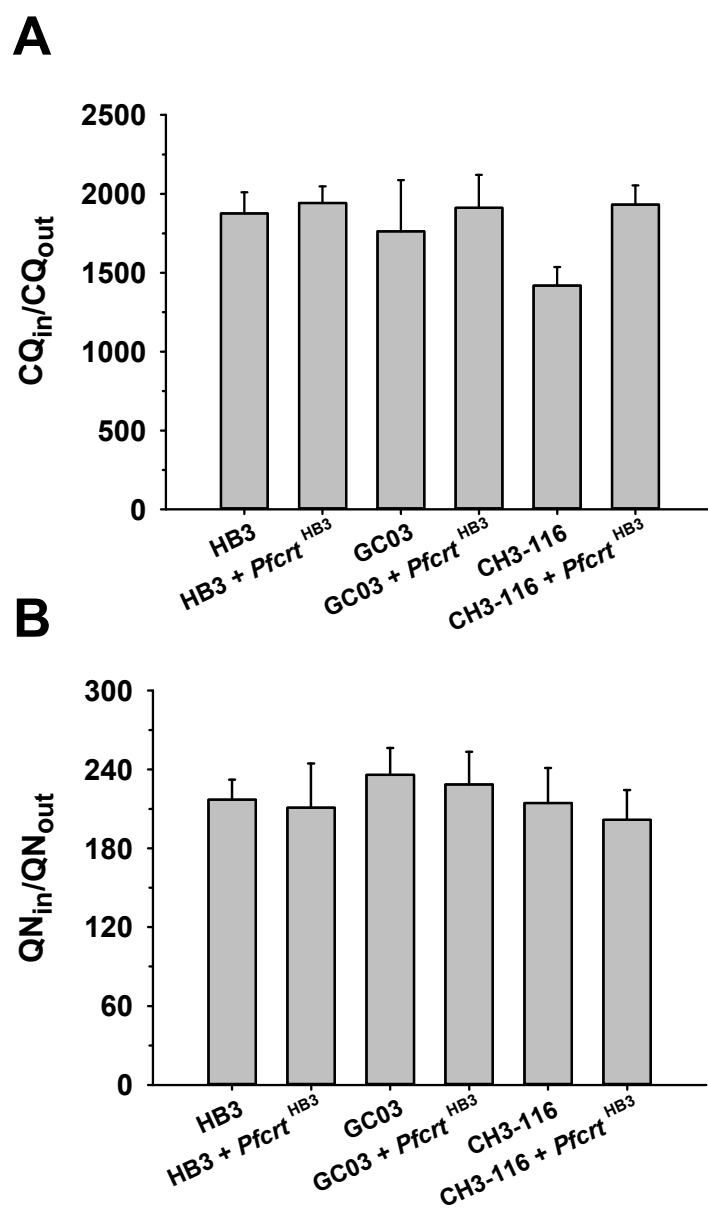

**Figure S5**

Supplement: Figure S5 — Effect of an episomally expressed wild type pfcrt allele on chloroquine (A) and quinine accumulation (B). Chloroquine and quinine accumulation levels were determined at the 20 min time point in transfected parasite lines and the corresponding parental strains. The means ± SEM of at least 10 independent determinations are shown. *, P<0.001. The genetic background of the parasite lines with regard to the genomic copy of pfcrt and the B5M12 locus are: HB3 and GCO3, pfcrt HB3 B5M12HB3; CH3-116, pfcrt HB3 B5M12Dd2. (PDF) [file pgen.1004382.s005.pdf]

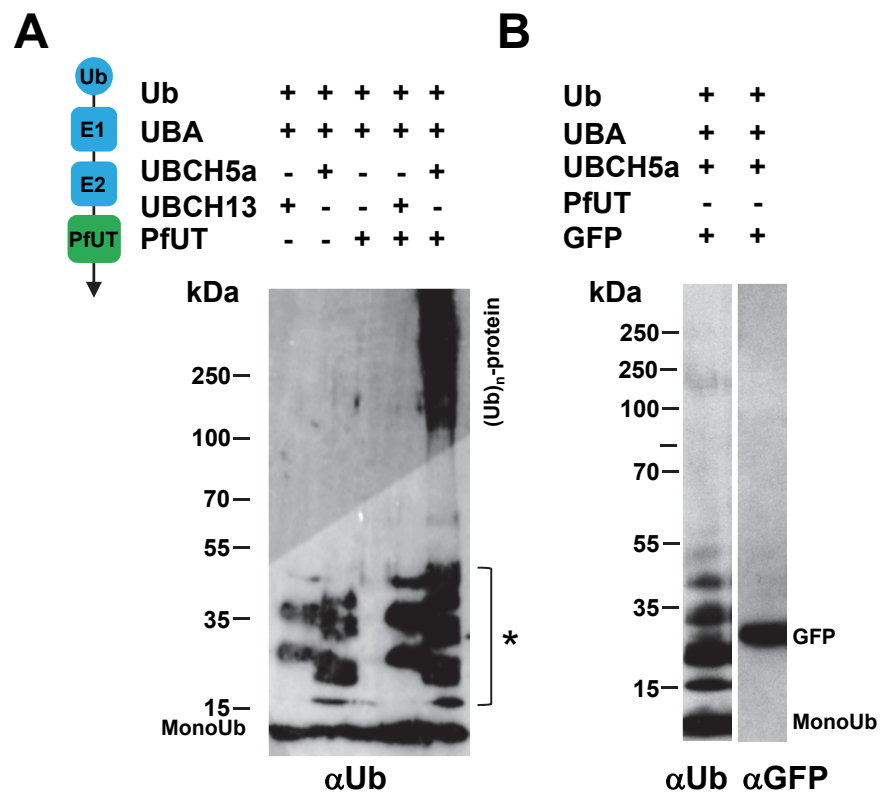

**Figure S6**

Supplement: Figure S6 — Parasite purified PfUT HECT domain/GFP fusion, but not GFP, catalyzes self polyubiquitination. A. The PfUT HECT domain/GFP fusion protein catalyzes self polyubiquitination. An independent biological replicate to the data presented in Figure 10A are shown. The PfUT HECT domain/GFP fusion protein was isolated from the corresponding transfected Dd2 line and the catalytic activity of the PfUT HECT domain/GFP fusion protein was tested in an in vitro assay reconstituted with the components indicated. The left scheme indicates the origin of the components and their function. The human components ubiquitin (Ub), E1 activating enzyme (E1; UBA), and the E2 conjugating enzymes (E2; UBCH5a or UBCH13) are highlighted in blue. The PfUT HECT domain/GFP fusion protein (PfUT) is indicated in green. The reactions were examined by Western analysis using SDS PAGE on a 4–12% gradient gel under non-reducing conditions and an antiserum specific to ubiquitin (αUb, dilution 1∶2000). The asterisk marks ubiquitin intermediate adducts generated by UBCH13 and UBCH5a. High molecular weight ubiquitinated products are indicated. A molecular weight marker is indicated in kDa. B. A parasite purified GFP is enzymatically inactive. Same experiment as described above, but this time parasite purified GFP was used. GFP was purified from a genetically engineered Dd2 line following the protocol established for the purification of PfUT HECT domain/GFP fusion. (PDF) [file pgen.1004382.s006.pdf]
