# Supplementary material for: A HECT Ubiquitin-Protein Ligase as a Novel Candidate Gene for Altered Quinine and Quinidine Responses in Plasmodium falciparum
Source: PLoS Genet. 2014 May 15;10(5):e1004382. doi: 10.1371/journal.pgen.1004382 (PMC4022464; doi:10.1371/journal.pgen.1004382)
Supplement: Table S4 — Compilation of relevant quantitative trait loci, LOD scores, and corresponding correlation coefficients. Only QTLs that rose above the confidence line of p<0.01 were considered. The chromosomal position of the markers in centi Morgan (cM) is indicated. A positive correlation coefficient (κ) indicates that the marker from Dd2 contributes to a decrease in drug susceptibility and an increase in drug accumulation. A negative correlation coefficient indicates that the marker from Dd2 contributes to an increase in drug susceptibility and a reduction in drug accumulation. QTLs identified in primary scans are highlighted in bold. QN, quinine; and QD, quinidine. (PDF) [file pgen.1004382.s010.pdf]

Table S4. Compilation of relevant quantitative trait loci, LOD scores, and corresponding correlation coefficients. Only QTLs that rose above the confidence line of p<0.01 were considered. The chromosomal position of the markers in centi Morgan (cM) is indicated. A positive correlation coefficient (κ) indicates that the marker from Dd2 contributes to a decrease in drug susceptibility and an increase in drug accumulation. A negative correlation coefficient indicates that the marker from Dd2 contributes to an increase in drug susceptibility and a reduction in drug accumulation. QTLs identified in primary scans are highlighted in bold. QN, quinine; and QD, quinidine.

Drug	Chr.	genetic distance (cM)	locus	accumulation (initial uptake)		accumulation (steady state)		QN IC_{90} or QD IC_{50}	
				LOD score	κ	LOD score	κ	LOD score	κ
QN	7	5.8	**B5M12**	6.6	- 0.74	4.9	-0.66	2.2	+0.47
	7	20.2	**pfcrt**	6.7	- 0.75	5.0	-0.67	3.0	+0.54
	13	164.5	**VAPA**	-	-	-	-	3.5	+0.58
	13	178.8	**C13M73**	-	-	-	-	3.7	+0.59
	5	69.9	MDR1*	2.0	+0.61	2.1	+0.64	-	-
	6	31.7	BM75*	-	-	-	-	2.6	+0.68
	6	100.5	BM103*	-	-	-	-	2.1	+0.68
	7	5.8	B5M12*	3.7	-0.79	2.6	-0.70	-	-
	13	32.6	MEF1*	3.0	-0.74	2.5	-0.69	-	-
	13	107.3	Poly3*	2.1	-0.64	-	-	-	-
	5	20.0	C5M2**	2.6	+0.66	2.1	+0.60	-	-
	6	23.1	B5M4**	-	-	-	-	2.0	-0.61
	13	178.8	C13M73**	-	-	-	-	3.1	+0.72
QD	6	51.7	**PF12**	2.2	0.47	-	-	-	-
	7	5.8	**B5M12**	5.8	-0.71	5.2	-0.68	3.2	+0.56

7	20.2	**pfcrt**	7.1	-0.76	4.7	-0.65	4.3	+0.63
13	107.3	**Poly3**	2.1	-0.46	2.0	-0.45	-	-
13	164.5	**VAPA**	-	-	-	-	2.8	+0.52
13	178.8	**C13M73**	-	-	-	-	2.1	+0.46
5	65.9	MDR1*	2.7	+0.71	2.5	+0.69	-	-
6	31.7	BM75*	-	-	-	-	2.0	+0.61
6	100.5	BM103*	-	-	-	-	2.3	+0.65
7	5.8	B5M12*	3.3	-0.76	3.0	-0.74	-	-
13	32.6	MEF1*	2.8	-0.70	2.5	-0.68	-	-
5	20	C5M2**	2.5	+0.65	-	-	-	-
9	0	C9M43**	2.0	+0.60	-	-	-	-
10	51.6	B7M14**	-	-	-	-	2.2	-0.62
11	60.3	AG15**	-	-	-	-	2.0	+0.60
14	9.6	C14M75**	-	-	2.8	+0.68	-	-
14	123.4	TP1**	2.4	-0.64	-	-	-	-

*   secondary scan: CQR only
** secondary scan: CQS only
